# N-terminus α-synuclein detection reveals new and more diverse aggregate morphologies in multiple system atrophy and Parkinson’s disease

**DOI:** 10.1186/s40035-024-00456-3

**Published:** 2024-12-27

**Authors:** James A. Wiseman, YuHong Fu, Richard L. M. Faull, Clinton P. Turner, Maurice A. Curtis, Glenda M. Halliday, Birger V. Dieriks

**Affiliations:** 1 Department of Anatomy and Medical Imaging, University of Auckland, 85 Park Road, Grafton, , Auckland, 1142 New Zealand; 2 Centre for Brain Research, University of Auckland, Auckland, 1023 New Zealand; 3 Brain and Mind Centre & Faculty of Medicine and Health School of Medical Sciences, The University of Sydney, Sydney, NSW 2050 Australia; 4 LabPlus, Department of Anatomical Pathology, Te Whatu Ora, Auckland, New Zealand; 5 Neuroscience Research Australia & Faculty of Medicine School of Medical Sciences, University of New South Wales, Sydney, NSW 2052 Australia

**Keywords:** α-Synuclein, Lewy body diseases, Parkinson’s disease, Multiple system atrophy, N-terminus, Epitope-specific, Multiplex, Truncational variants

## Abstract

**Background:**

Parkinson’s disease (PD) and multiple system atrophy (MSA) are classified as α-synucleinopathies and are primarily differentiated by their clinical phenotypes. Delineating these diseases based on their specific α-synuclein (α-Syn) proteoform pathologies is crucial for accurate antemortem biomarker diagnosis. Newly identified α-Syn pathologies in PD raise questions about whether MSA exhibits a similar diversity. This prompted the need for a comparative study focusing on α-Syn epitope-specific immunoreactivities in both diseases, which could clarify the extent of pathological overlap and diversity, and guide more accurate biomarker development.

**Methods:**

We utilised a multiplex immunohistochemical approach to detect multiple structural domains of α-Syn proteoforms across multiple regions prone to pathological accumulation in MSA (*n* = 10) and PD (*n* = 10). Comparison of epitope-specific α-Syn proteoforms was performed in the MSA medulla, inferior olivary nucleus, substantia nigra, hippocampus, and cerebellum, and in the PD olfactory bulb, medulla, substantia nigra, hippocampus, and entorhinal cortex.

**Results:**

N-terminus and C-terminus antibodies detected significantly more α-Syn pathology in MSA than antibodies for phosphorylated (pS129) α-Syn, which are classically used to detect α-Syn. Importantly, C-terminus immunolabelling is more pronounced in MSA compared to PD. Meanwhile, N-terminus immunolabelling consistently detected the highest percentage of α-Syn across pathologically burdened regions of both diseases, which could be of biological significance. As expected, oligodendroglial involvement distinguished MSA from PD, but in contrast to PD, no substantial astrocytic or microglial α-Syn accumulation in MSA occurred. These data confirm glial-specific changes between these diseases when immunolabelling the N-terminus epitope. In comparison, N-terminus neuronal α-Syn was present in PD and MSA, with most MSA neurons lacking pS129 α-Syn proteoforms. This explains why characterisation of neuronal MSA pathologies is lacking and challenges the reliance on pS129 antibodies for the accurate quantification of α-Syn pathological load across α-synucleinopathies.

**Conclusions:**

These findings underscore the necessity of utilising a multiplex approach to detect α-Syn, most importantly including the N-terminus, to capture the entire spectrum of α-Syn proteoforms in α-synucleinopathies. The data provide novel insights toward the biological differentiation of these α-synucleinopathies and pave the way for more refined antemortem diagnostic methods to facilitate early identification and intervention of these neurodegenerative diseases.

**Supplementary Information:**

The online version contains supplementary material available at 10.1186/s40035-024-00456-3.

## Introduction

Both Parkinson’s disease (PD) and multiple system atrophy (MSA) have α-synuclein (α-Syn) aggregates now known to be different in their proteoform structure, with MSA having multiple types of structures identified [1–4]. PD and MSA are currently diagnosed on their differentiating clinical features [5, 6], but this is about to change with a push to identify these patients in life according to their underlying α-Syn pathology [7, 8]. This has particular significance for those with early or prodromal disease where clinical features are much less certain but where there is a known accumulation of α-Syn in discrete brain regions [9–12]. Understanding the differences in brain α-Syn proteoforms is now paramount. We recently identified more α-Syn aggregates in post-mortem PD cases when using diverse α-Syn antibodies [13], adding to the literature of more pathologic accumulations than previously thought in both PD [14–25] and MSA [17, 18, 20, 21, 26–28]. There have been much more limited studies comparing different proteoforms between PD and MSA [17, 18, 20], despite the importance of identifying these differences to increase the potential to biologically identify these diseases in life.

**Fig. 1:**
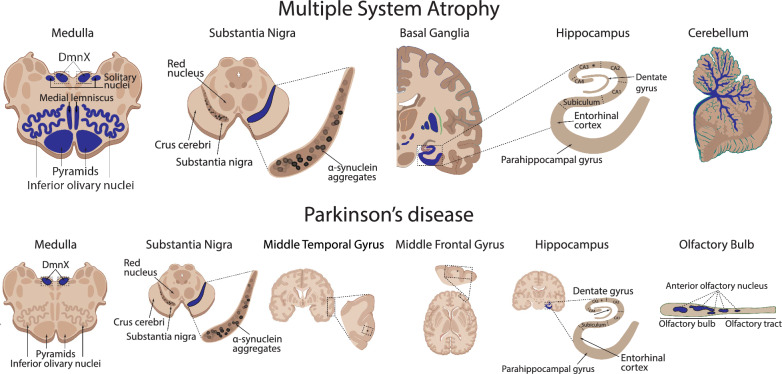
Comparison of the regional distribution of α-Syn pathology in the human brain with MSA and PD. Schematic diagram depicting the anatomical localisation (blue) of α-Syn pathology in the medulla, substantia nigra, hippocampus, and cerebellum of the human brain with MSA *(top)*, and the medulla, substantia nigra, middle temporal gyrus, middle frontal gyrus, hippocampus, and olfactory bulb of the human brain with PD *(bottom)*

The main α-Syn pathologies used to differentiate MSA from PD are glial cytoplasmic inclusions (GCIs) for MSA and neuronal Lewy pathology (LP) for PD, which have different regional distributions in the brain (Fig. 1) [29]. However, MSA also has considerable neuronal α-Syn accumulations that have been understudied [27, 30, 31]. The neuronal inclusions in MSA are largely composed of loosely packed α-Syn filaments, and they have additional neuronal intranuclear inclusions that are not observed in PD [32–34]. The proteoforms of these MSA neuronal inclusions have not been characterised in detail, and only a handful of studies have investigated the abundance of α-Syn truncational variants in MSA [35–37]. The marked loss of non-dopaminergic neurons in MSA precipitates two disease subtypes, which are clinically diagnosed based on the dominant clinical symptomatology. Neuronal loss within the striatonigral regions typically manifests as parkinsonism (MSA-P) when dominant, whereas neuronal loss within the olivopontocerebellar regions typically manifests as cerebellar ataxia (MSA-C), with both subtypes exhibiting a variable combination of autonomic dysfunctions [38–41].

Our recent publication demonstrating the diversity of α-Syn proteoforms in PD [13] prompted this comparative investigation of the human brain with MSA. In this study we utilise the same antibody panel to compare multiregional epitope-specific α-Syn aggregates between MSA and PD to (1) investigate if α-Syn aggregates in MSA exhibit differential epitope-specific immunoreactivities across aggregate-prone regions, (2) determine whether any region-specific aggregate morphologies manifest in the human brain with MSA compared to PD, and (3) to quantify the extent of epitope-specific α-Syn detection across aggregate-prone regions in MSA and directly compare this to PD.

## Methods and materials

### Study design and statistics

We recently utilised multiplex immunohistochemistry – with antibodies mapping the three structural domains of α-Syn – to simultaneously immunolabel tissue sections from multiple pathologically burdened regions of the brains of PD patients [13]. No pathological staining was observed in neurologically normal controls with any of the α-Syn antibodies [13]. Importantly, immunoreactivity of the N-terminus revealed previously uncharacterised populations of (1) perinuclear, (2) glial (microglial and astrocytic), and (3) neuronal lysosomal α-Syn aggregates in the human PD brain [13]. While similar studies have been conducted that interrogate the epitope-specific diversity of α-Syn in Lewy body diseases [14, 15, 19, 20, 42], no studies have investigated the epitope-specific construct of different α-Syn proteoforms in the human brains with MSA using multiplex immunohistochemistry, nor compared the epitope-specific distribution of α-Syn proteoforms between MSA and PD.

Comparison of epitope-specific α-Syn proteoforms was performed in the MSA (*n* = 10) medulla, inferior olivary nucleus, substantia nigra, hippocampus, and cerebellum, and the PD (*n* = 10) olfactory bulb, medulla, substantia nigra, hippocampus, and entorhinal cortex (Table 1, Table S1). Cohorts, tissue preparation, procedures of formalin-fixed paraffin-embedded tissue processing, and procedure of fluorescent immunohistochemistry are provided in Supplementary Methods. Sections were multiplex immunolabelled with a previously described panel of epitope-specific α-Syn antibodies [13]. The α-synuclein A15110D antibody (Catalogue No. 849102, BioLegend, San Diego, CA) detects residues 34–45 in the N-terminus. The α-synuclein (81A) antibody (Catalogue No. Ab184674, Abcam, Cambridge, United Kingdom) detects phosphorylated serine 129. The α-synuclein MJFR1 antibody (Catalogue No. Ab138501, Abcam) detects residues 118–123 in the C-terminus (Tables S2 and S3). MAP2 and p25α/TPPP were used to ascertain the neuronal- and oligodendroglial-specific localisation of α-Syn inclusions, respectively. Whole-section slide scans and representative confocal images were acquired.

**Table 1:** Summary of case statistics (Mean ± SD) for human brain tissue used in this study (see Supplementary materials for further information)

	MSA	PD	Neurologically normal
*n*	10 (MSA-C, *n* = 5; MSA-P, *n* = 5)	10	5
Disease duration (years)	10.1 ± 5.1	17.1 ± 6.6	–
Age (years)	67.7 ± 8.5	74.9 ± 5.3	81.8 ± 11.6
PMD (hours)	24.6 ± 11.6	12.3 ± 7.1	26.2 ± 7
Average ABC Score [43] (maximum = 3)	A 0.5 ± 0.2 B 0.4 ± 0.2 C 0	A 1.4 ± 1.2 B 0.6 ± 0.7 C 0.8 ± 0.8	A 0.7 ± 0.3 B 1.0 ± 0.6 C 0
Braak Lewy Pathology Stage [10] (maximum = 6)	0	4.9 ± 0.8	0
MSA Grade [44] (maximum = 3)	SND 2.1 ± 0.3 OPCA 1.3 ± 0.3	0	0

ABC score: beta-amyloid plaque stage (A), Braak neurofibrillary tangle stage (B), and CERAD neuritic plaque severity (C); MSA, multiple system atrophy; MSA-C, MSA-cerebellar; MSA-P, MSA-parkinsonism; OPCA, olivopontocerebellar atrophy; PD, Parkinson’s disease; PMD, post-mortem delay; SND, striatonigral degeneration

To quantify differential α-Syn epitope immunolabelling in α-Syn aggregates, three distinct quantification methods were utilised, which are graphically summarised in Fig. S1. First, the *total* α-Syn immunolabelling area was determined. This was defined as the combined α-Syn immunolabelling area of all α-Syn antibodies. The total α-Syn immunolabelling area was determined by calculating the net α-Syn immunolabelling across the N-terminus (849102), pS129 (Ab184674), and C-terminus (Ab138501) antibodies (see graphical representation of quantification in Fig. S1). Second, the total immunolabelling area for each *individual* epitope was determined as a percentage of the total α-Syn immunolabelling area for an individual α-Syn antibody (including overlapping area with other α-Syn antibodies) relative to the total α-syn immunolabeling area. Finally, the *unique* epitope-specific immunolabelling of each antibody was defined as pathological α-Syn that was exclusively detected by a single α-Syn antibody (Fig. S1), and determined as a percentage of the total α-Syn immunolabelling area.

Data visualisation and statistical analysis were performed using RStudio (R version 4.3.1). All data are presented as mean ± SD. The Shapiro–Wilk test was used to assess data distribution. Parametric tests were utilised when data were normally distributed, and non-parametric tests were performed when data did not assume a normal distribution. Linear regression, using Pearson’s correlation coefficient, was used to analyse correlations between epitope-specific α-Syn antibody labelling. A repeated-measures one-way ANOVA was used to compare differential epitope-specific α-Syn immunoreactivities across interrogated brain regions, with Tukey’s multiple comparison adjustment. When comparing the epitope-specific immunolabelling between PD and MSA, unpaired *t*-tests or the Mann–Whitney U test were used. Statistical significance was set as *P* < 0.05.

### Image acquisition and processing

Confocal images were acquired using an LSM 800 with Airyscan confocal microscope (Zeiss, Oberkochen, Germany) with a 63x/1.4 NA Plan Apochromat DIC M27 oil immersion objective lens and GaAsp-PMT detector. Images were acquired using the built-in Airyscan module and processed using the ZEN microscopy software (Zeiss). All images were acquired using optimal Nyquist sampling parameters, and those acquired in a Z-series used the optimal step size of 0.13 μm. Images were acquired from the medulla, the substantia nigra pars compacta, the CA2 region of the hippocampus proper, the entorhinal cortex, and the cerebellar white matter. Stimulated emission depletion (STED) imaging was performed on a Leica TCS SP8 microscope (Leica Microsystems GmbH, Mannheim, Germany), using a 93 × 1.30 NA glycerol immersion motCORR STED White objective and a tuneable white light laser unit. All deconvolution of STED images and 3D reconstruction of aggregates were performed using the Huygens Professional software package (Scientific Volume Imaging, Hilversum, The Netherlands).

Whole-tissue images were acquired using an automated fluorescence microscope (Zeiss Axioimager Z2) equipped with a MetaSystems VSlide slide scanner (MetaSystems) and Colibri 7 light source, running Metafer 5 (v4.4.114) with a Plan-Apochromat 20x/0.8 NA dry objective lens. Images were stitched using the MetaCyte software and exported in .IMS format so they were compatible with QuPath. Following image acquisition, the total section scans were viewed in QuPath and specific regions for quantification were identified and exported in OME TIFF format for subsequent analysis in FIJI. All downstream quantitative image analysis was performed using FIJI/ImageJ (V 2.14.0/1.54f, Bethesda, MA). Briefly, background signal intensity was subtracted using an automated Gaussian blur algorithm, and pathological α-Syn labelling was automatically thresholded using the Otsu algorithm for each epitope-specific antibody. This thresholding approach for α-Syn segmentation ensured that all quantifications were specific to pathological α-Syn, as only defined α-Syn aggregates with threshold intensities above that of non-pathological monomeric α-Syn and non-specific background signatures (observed in neurologically normal cases) were captured in the thresholding process and subsequently quantified. Accurate aggregate segmentation was manually confirmed prior to conducting downstream analysis. Representative images displaying this segmentation process in whole-section scans and confocal acquisitions are displayed in Fig. S2.

## Results

### Types of α-synuclein inclusion pathologies in MSA compared with PD

Table 2 summarises previous studies and the current comparative data using α-Syn antibodies that target different epitopes in MSA and PD. Most previous studies have been on PD and used epitopes within the C-terminus of α-Syn, including pS129. Despite being susceptible to C-terminal truncation and post-translational modification, detection of C-terminus epitopes generally identifies classical LP in PD (Table 2), and we recently identified a novel population of neuritic α-Syn proteoforms that exhibit exclusive pS129 immunoreactivity in PD (Table 2, novel PD pathology) [13]. In addition to these neuronal pathologies, several studies have shown the detection of astrocytic α-Syn proteoforms with C-terminus antibodies, but N-terminus immunolabelling detects more abundant populations of astrocytic α-Syn proteoforms that are not detected with C-terminus or pS129 antibodies (Table 2). Moreover, immunolabelling of the N-terminus (residues 34-57) and NAC domain (residues 80-96—detected by A15115A antibody, BioLegend) reveals novel populations of neuronal lysosomal, microglial, and perinuclear α-Syn proteoforms (Table 2) [13].

**Table 2:** Summary of literature and overview of novel findings

		C-terminus	pS129	N-terminus
PD Neuronal Pathology	Previously shown	LB and LN pathology, threads [14–18] Dot-like pathology [21, 22]	LB and LN pathology, threads [14, 16, 18, 23] Dot-like pathology [21]	LB and LN pathology, threads [14, 18–20] Dot-like pathology [22]
	Novel finding by Wiseman et al. (2024) [13]	–	Neuritic α-Syn proteoforms with exclusive pS129 immunoreactivity [13]	Punctate lysosomal proteoforms [13] Perinuclear proteoforms [13]
PD Glial Pathology	Previously shown	Astrocytic proteoforms [16, 24, 25, 45]	–	Astrocytic proteoforms [19, 20, 24, 25]
	Novel finding by Wiseman et al*.* (2024) [13]	–	–	Microglial and astrocytic proteoforms [13]
MSA Neuronal pathology	Previously shown	NCIs, intranuclear inclusions, threads [17, 21, 26, 27]	NCIs, intranuclear inclusions, dot-like inclusions, threads [18, 21]	NCIs, intranuclear inclusions, dot-like inclusions, threads [20, 26]
	Novel finding in this study	Filamentous NCI proteoforms with unique C-terminus immunoreactivity	pS129 fails to detect a significant proportion of NCIs	Filamentous NCI proteoforms with unique N-terminus immunoreactivity
MSA Glial pathology	Previously shown	GCIs, intranuclear inclusions [17, 26]	GCIs [18, 21, 28]	GCIs [20, 26]
	Novel finding in this study	Filamentous GCI proteoforms with unique C-terminus immunoreactivity	pS129 fails to detect a significant proportion of GCIs	GCIs, intranuclear inclusions. Filamentous GCI proteoforms with unique N-terminus immunoreactivity
Neurologically normal		Low level of punctate immunoreactivity in cytoplasm [13, 46]	No presence of pS129 α-Syn [13, 46]	Low level of punctate immunoreactivity in cytoplasm revealed in previous studies [13, 46] and in this study

GCI, glial cytoplasmic inclusion; NCI, neuronal cytoplasmic inclusion; LB, Lewy body; LN, Lewy neurite

**Fig. 2:**
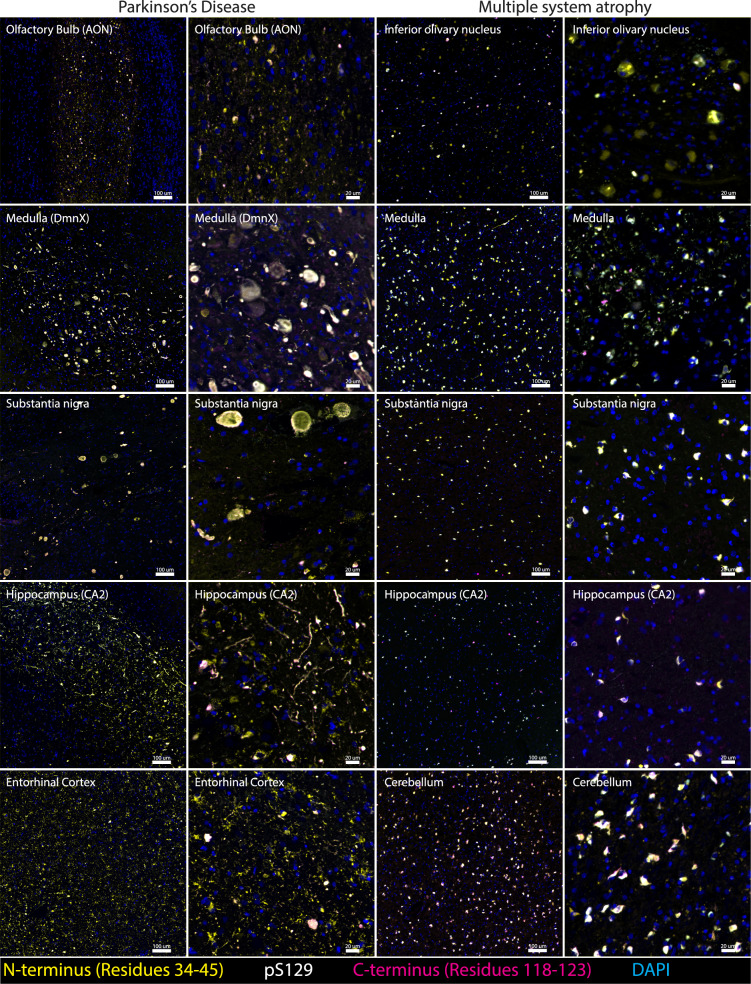
Representative images demonstrating the regional distribution and epitope-specific immunolabelling of α-Syn aggregates in PD (left) and MSA (right). Overview images of the whole regions are displayed in the left column for each disease (Scale bars, 100 μm), and zoomed overview images for each region are displayed in the right column for each disease (Scale bars, 20 μm)

**Fig. 3:**
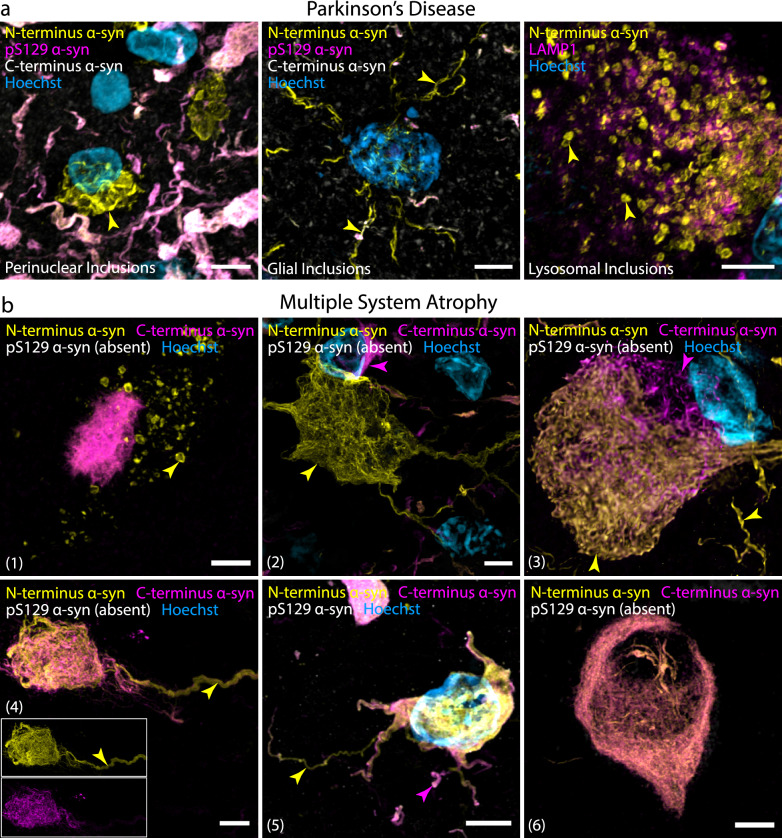
Representative confocal images showing different epitope-specific aggregate structures found in PD and MSA. **a** Perinuclear, glial, and lysosomal α-Syn aggregates with exclusive N-terminus immunoreactivity in the human brain with PD. **b** Different epitope-specific aggregate structures found in MSA*.* (1) Punctate aggregates exhibiting exclusive N-terminus immunoreactivity were commonly observed within neuronal cytoplasm. (2) Neuronal inclusions with exclusive N-terminus immunoreactivity were frequently observed in the inferior olivary nucleus*.* (3, 4) Filamentous neuronal inclusions with exclusive portions of N-terminus immunoreactivity and exclusive portions of C-terminus immunoreactivity were common. (5) Oligodendroglial inclusion exhibiting variable N-terminus, pS129, and C-terminus immunoreactivity. (1–4, 6) pS129 α-Syn immunolabelling was often very weak or, in some instances, completely absent. Yellow and magenta arrowheads label instances of unique N-terminus and C-terminus immunolabelling, respectively. Scale bars, 5 μm

**Fig. 4:**
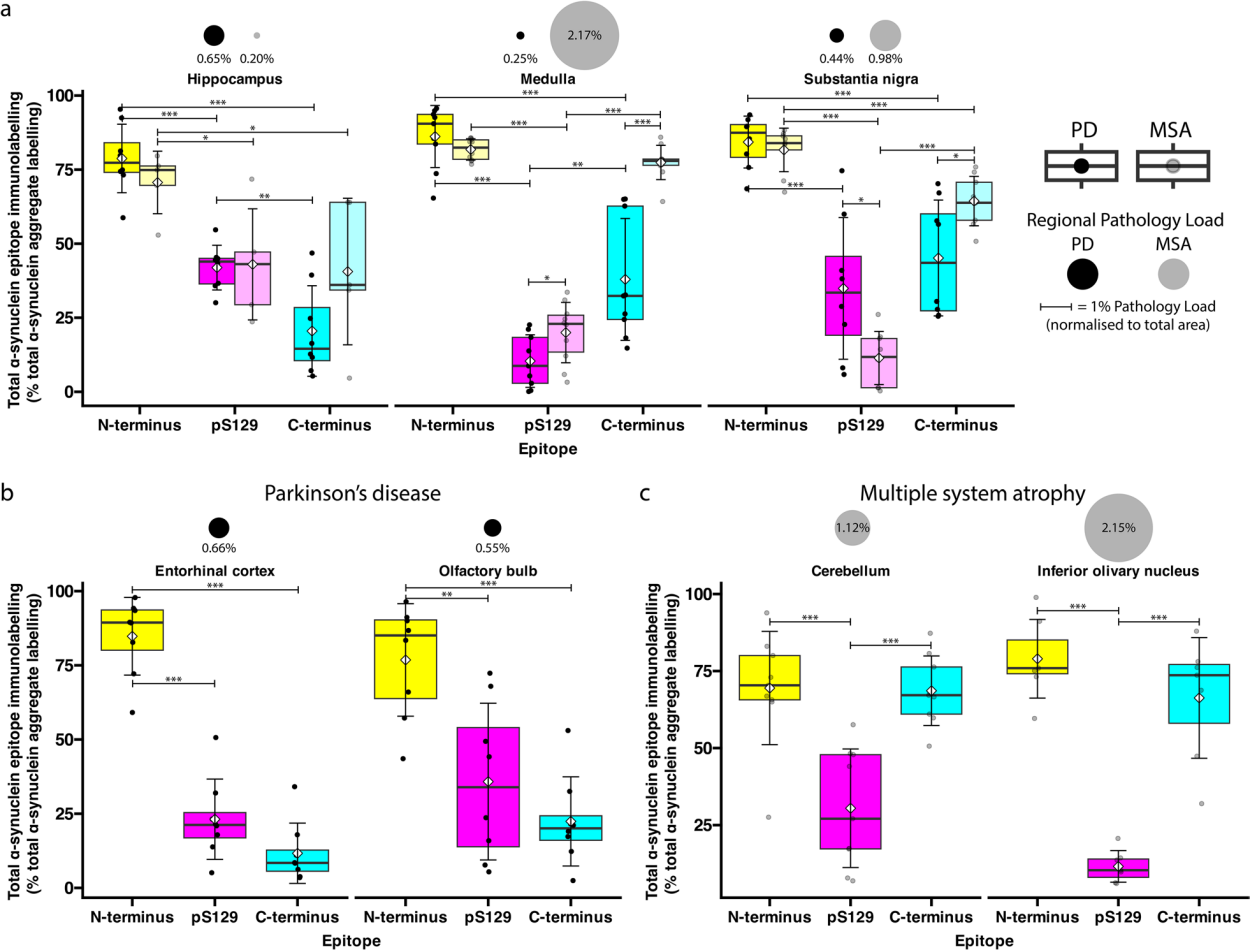
Comparison of total individual α-synuclein epitope immunolabelling in PD and MSA. Quantification of total N-terminus (yellow), pS129 (magenta), and C-terminus (cyan) epitope immunolabelling in (**a**) the hippocampus, medulla, and substantia nigra of PD and MSA cases, (**b**) the PD entorhinal cortex and olfactory bulb, and (**c**) the MSA cerebellum and inferior olivary nucleus. Boxplot colours correspond to colours of N-terminus (yellow), pS129 (magenta), and C-terminus (cyan) immunolabelling in representative images. White diamond = mean; crossbar = median; boxplots display interquartile range, and error bars indicate SD. ***P < 0.001, **P < 0.01, *P < 0.05

Though the study of epitope-specific α-Syn proteoforms in MSA is less established than that in PD, C-terminus, pS129, and N-terminus antibodies have all been shown to detect classical GCIs, neuronal cytoplasmic and neuronal intranuclear inclusion pathologies in MSA (Table 2), although all previous studies have relied on single-staining methodologies (which precludes reliable comparisons of detection efficacy between different antibodies and the spatial resolution of discrete versus overlayed α-Syn epitopes). N-terminus polymorphs that exhibit a perinuclear, microglial, or astrocytic disposition, which we have previously observed in PD [13], were entirely absent in MSA (Fig. 2, PD Entorhinal cortex; Fig. 3a). In contrast, we frequently observed punctate aggregates within the neuronal cytoplasm that exhibited exclusive N-terminus immunoreactivity in MSA (Fig. 3b(1)), albeit to a much lesser extent than that in PD (Figs. 3 and 4; Video S1). N-terminus and C-terminus immunolabelling reliably detected all hallmark MSA α-Syn pathologies (Figs. S3, S4, S5, and S6). In comparison, pS129 immunolabelling was relatively poor, often with completely absent staining (Figs. 3, S5, and S6). Strikingly, within the inferior olivary nucleus (ION) and adjacent medullary tracts of MSA, there were dense populations of neuronal inclusions that were exclusively N-terminus-immunoreactive (Fig. 3b(2); Videos S2 and 3). These large filamentous MSA inclusions typically infiltrate the entire neuronal cell body and spread to occupy the dendrites, axon hillock, and axon. With exception of these N-terminus^+^ neuronal inclusions in the ION, C-terminus immunolabelling reliably detected all inclusion types identified in MSA.

High-resolution confocal and STED microscopy identified several filamentous neuronal inclusions in which whole portions of the total aggregate construct were exclusively N-terminus immunoreactive (yellow) or exclusively C-terminus immunoreactive (magenta), with the remainder of the aggregate exhibiting combined immunoreactivity (Fig. 3b(3); Video S4). In addition, discrete whisp-like α-Syn filaments with either exclusive N-terminus or exclusive C-terminus immunoreactivity were frequently observed, which appeared to be interlaced and juxtaposed with one another (Fig. 3b(4); Fig. S7a; Profile Video S1). pS129 detection was not observed in the filamentous inclusions.

The demarcation of α-Syn inclusion boundaries in MSA is dictated by the structural confines of the intracellular space, unlike LP in PD. Oligodendroglial inclusions were remarkably heterogeneous, both within and across all investigated regions (Figs. S3 and S4, Cerebellum). This heterogeneity arises from the expansive cytoarchitectural diversity of oligodendrocytes within the human brain. In healthy human brains, this structural heterogeneity among oligodendroglia is not immediately obvious due to their relatively small soma. In MSA, however, accumulation of filamentous α-Syn within oligodendroglia causes somatic enlargement, making this cytoarchitectural diversity more apparent. Oligodendroglial α-Syn inclusions were almost exclusively intracellular, manifesting as dense, irregularly shaped clusters, typically surrounding the cellular nucleus (Figs. S3, S4, and S7b-d). This morphological irregularity, nuclear-centric arrangement and exclusive intercellular disposition of α-Syn inclusions in MSA is distinct from the LP typically found in PD. Surprisingly, no notable region-specific inclusion morphologies were observed in MSA, with the exception of the filamentous morphology of neuronal inclusions in the ION and, to a lesser extent, the surrounding medullary tracts (Figs. 2, 3b, S3 and S4). Similar to oligodendroglial inclusions, neuronal ION inclusions were morphologically defined by the neuronal periphery. Figure S8 highlights the structures of a typical oligodendroglial (top) and a typical neuronal (bottom) inclusion in MSA, and highlights the morphological demarcation of their inclusion boundaries by the cellular periphery.

Neuronal inclusions were predominantly composed of loosely intertwined filamentous α-Syn, reminiscent of a web-like structure (Figs. 3b, S3, S4, S5a). These filamentous inclusions typically spread throughout the neuronal intracellular space, not only infiltrating the neuronal nucleus and cytoplasm but also spreading through to the dendritic process, the axon hillock and axon (Fig. 3b, S3, S4, and S8, Neuronal Inclusion). The cellular distribution of α-Syn inclusions in the medulla, substantia nigra, and hippocampus was highly mixed between neurons and oligodendrocytes, with the exception of the ION, where almost no GCIs were observed. Across regions, however, GCIs were consistently observed in higher proportions compared to neuronal inclusions. Neuronal inclusions within the cerebellum were sparse, with almost all inclusions being GCIs (Fig. 2, MSA; Figs. S3, S4).

Throughout all investigated regions, GCIs that infiltrate the entire intracellular space and spread to the glial processes were observed (Figs. 3b(5), S3, S4, S6; Supplementary Video S5). Typically, these inclusions were composed of interlacing α-Syn filaments that were relatively compact but still structurally discrete. Similar to those constituting neuronal inclusions, these oligodendroglial α-Syn filaments also exhibited differential epitope-specific immunoreactivity (Fig. S7b-d, Video S6, Profile Videos S3-S5). These dense and structurally well-defined GCIs were typically characterised by a distinct C-terminus immunopositive periphery that appeared to be encapsulating inclusions, analogous to that of peripheral phosphorylated α-Syn, which is observed in LP in PD [14] (Fig. S3–S5; Video S7; Profile Videos S2-S4).

### Regional quantitation of α-Syn pathologies using multi-epitope detection in MSA and PD

As anticipated, the regional distribution of α-Syn pathology in MSA differed substantially from that observed in PD. α-Syn pathology in the medulla was diffuse across the entire region, with abundant pathology present in the ION (Fig. 2, MSA), the pyramids, the medial lemniscus, the solitary nuclei, the raphe nuclei, and the reticular formation (Fig. S9), as also shown by Cykowski et al*.* (2015) [31]. These are regions that are generally spared in PD. In the midbrain, α-Syn pathology was prominent in the substantia nigra pars compacta, with some pathology also present within immediately adjacent midbrain and nigral structures, including the substantia nigra pars reticularis (Fig. 2, MSA). α-Syn pathology in the hippocampus was relatively sparse, which is in stark contrast to PD, where a dense band of α-Syn pathology is usually present in the CA2 subregion of the hippocampus proper (Fig. 2, PD Hippocampus). Moreover, α-Syn pathology was also relatively sparse within adjacent regions to the hippocampus in MSA, namely the subiculum, entorhinal cortex, parahippocampal gyrus, and fusiform gyrus, which exhibit relatively high pathological burden in PD. In contrast, cerebellar pathology was profuse and almost entirely restricted to the central white matter tracts (Fig. 2, MSA), with an overwhelming proportion of inclusions localised to oligodendroglia.

Quantification of α-Syn pathology load revealed stark regional differences between PD and MSA (Fig. 4). The pathology load was defined as the percentage of total α-Syn immunolabelling area (the combined immunolabelling of the N-terminus, pS129, and C-terminus α-Syn antibodies) to the tissue area. In PD, the mean pathology load in the hippocampus was 0.65% ± 0.51% (mostly N-terminus, Table S4), which was threefold higher than that in MSA (Fig. 4). In contrast, the mean pathology load in the PD medulla was relatively low at 0.25% ± 0.07%, whilst the mean pathology load in the MSA medulla was significantly higher (*P* < 0.001)*,* the highest of all interrogated regions in MSA (both N- and C-terminus, Fig. 4; Table S4). In the substantia nigra, the mean pathology load in PD was 0.45% ± 0.18%, whilst it was 0.99% ± 0.54% in MSA (mostly N-terminus, *P* < 0.05; Table S4). Within the entorhinal cortex and olfactory bulb in PD, the mean pathology load was 0.66% ± 0.30% and 0.55% ± 0.28%, respectively (mostly N-terminus). In comparison, no quantifiable α-Syn pathology was present within the entorhinal cortex in MSA cases. Within the cerebellum and ION of MSA cases, the mean pathology loads were 1.12% ± 0.69% and 2.15% ± 1.29% (mostly N- and C-terminus), which were significantly higher than in PD where these regions were spared. There was no significant difference in pathology load between PD regions, with the exception of the entorhinal cortex having significantly higher pathology than the medulla (*P* < 0.05). In contrast, the mean pathology load was variable across different MSA regions. In particular, the mean pathology load within the MSA hippocampus was significantly lower than that in the MSA medulla, substantia nigra, cerebellum, and ION (*P* < 0.05). In addition, the MSA ION and medulla both had significantly more α-Syn pathology than the MSA substantia nigra (*P* < 0.05).

### α-Syn proteoforms differ between MSA and PD

**Fig. 5:**
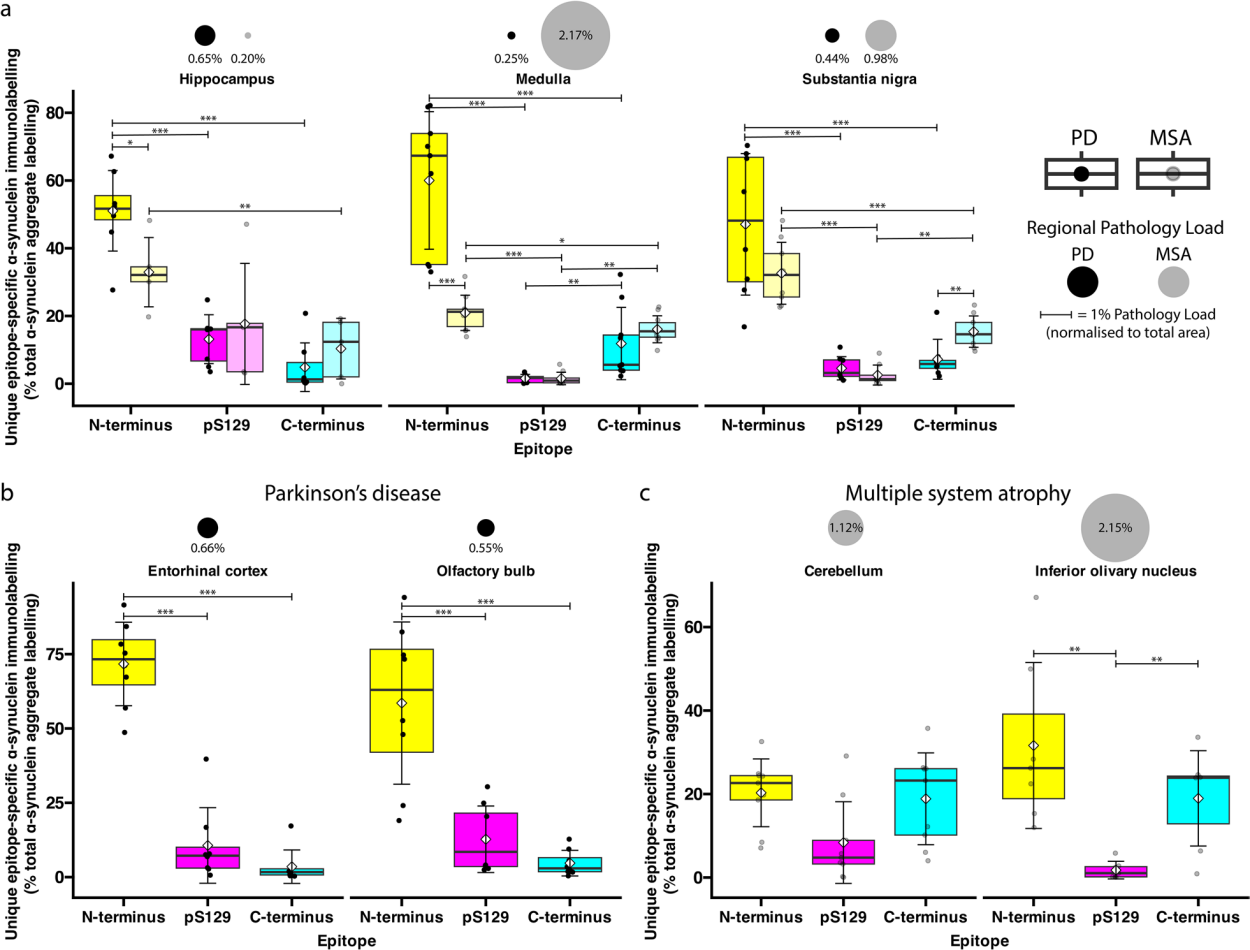
Comparison of unique (epitope-specific) α-synuclein immunolabelling in PD and MSA. Quantification of unique (epitope-specific) N-terminus (yellow), pS129 (magenta), and C-terminus (cyan) immunolabelling in (**a**) the hippocampus, medulla, and substantia nigra of PD and MSA cases, (**b**) the PD entorhinal cortex and olfactory bulb, and (**c**) the MSA cerebellum and inferior olivary nucleus. Boxplot colours correspond to colours of N-terminus (yellow), pS129 (magenta), and C-terminus (cyan) immunolabelling in representative images. White diamond = mean; crossbar = median; boxplots display interquartile range, and error bars indicate SD. ***P < 0.001, **P < 0.01, *P < 0.05

To quantify unique epitope-specific α-Syn immunolabelling in PD and MSA, pathological α-Syn that was exclusively detected by a single α-Syn antibody was identified. In PD, the mean unique epitope-specific immunolabelling of N-terminus, pS129, and C-terminus antibodies, respectively, was 51.05% ± 11.89%, 13.15% ± 7.22%, and 4.87% ± 7.17% of the total α-Syn immunolabelling area in the hippocampus, 60.01% ± 20.31%, 1.57% ± 1.17%, and 11.85% ± 10.68% in the medulla, and 47.06% ± 20.90%, 4.55% ± 3.43%, and 7.21% ± 5.90% in the substantia nigra (Fig. 5a). In the PD entorhinal cortex, the mean epitope-specific immunolabelling of N-terminus, pS129, and C-terminus antibodies, respectively, was 71.70% ± 14.04%, 10.65% ± 12.72%, and 3.50% ± 5.63%, respectively; in the PD olfactory bulb, the mean epitope-specific immunolabelling was 58.54 ± 27.29%, 12.72 ± 11.19%, and 4.69 ± 4.32%, respectively (Fig. 5b). The mean epitope-specific N-terminus immunolabelling was significantly higher than the pS129 and C-terminus immunolabelling across all interrogated regions (*P* < 0.001).

In MSA, the mean unique epitope-specific immunolabelling of N-terminus, pS129, and C-terminus antibodies, respectively, was 32.94% ± 10.23%, 17.66% ± 17.87%, and 10.35% ± 8.94% in the hippocampus, 20.91% ± 5.21%, 1.54% ± 1.84%, and 16.07% ± 3.99% in the medulla, and 32.60% ± 9.14%, 2.56% ± 2.95%, and 15.38% ± 4.62% in the substantia nigra (Fig. 5a). In the MSA cerebellum, the mean epitope-specific immunolabelling for N-terminus, pS129, and C-terminus antibodies was 20.29% ± 8.12%, 8.37% ± 9.79%, and 18.86% ± 11.00%, respectively; and that in the MSA ION was 31.63% ± 19.88%, 1.77% ± 2.11%, and 18.96% ± 11.42%, respectively (Fig. 5c). In MSA, the mean epitope-specific N-terminus immunolabelling was significantly higher than the pS129 immunolabelling in the medulla (*P* < 0.001), substantia nigra (*P* < 0.001) and ION (*P* < 0.01), and the C-terminus immunolabelling in the hippocampus (*P* < 0.01), medulla (*P* < 0.05), and substantia nigra (*P* < 0.001; Fig. 5). Table S5 displays the mean total and mean unique epitope-specific immunolabelling of the N-terminus, pS129, and C-terminus antibodies across all regions for both PD and MSA.

Interestingly, in PD, the unique epitope-specific N-terminus and pS129 immunoreactivity exhibited a strong negative correlation in the olfactory bulb (*r* =  − 0.957, *P* < 0.001; Fig. S10a). The total N-terminus and pS129 immunoreactivity also exhibited a strong negative correlation in the olfactory bulb (*r* =  − 0.910, *P* < 0.005; Fig. S10b), substantia nigra (− 0.787, *P* < 0.05; Fig. S10e), and entorhinal cortex (− 0.856, *P* < 0.01; Fig. S10g). The total N-terminus and C-terminus immunoreactivity exhibited a strong negative correlation in the olfactory bulb (− 0.915, *P* < 0.005; Fig. S10c), substantia nigra (− 0.738, *P* < 0.05; Fig. S10f), and medulla (− 0.683, *P* < 0.05; Fig. S10h). Finally, the total pS129 and C-terminus immunolabelling exhibited a strong positive correlation in the olfactory bulb (0.851, *P* < 0.01; Fig. S10d).

In MSA, the unique epitope-specific N-terminus and C-terminus immunoreactivity exhibited a strong negative correlation in the cerebellum (− 0.718, *P* < 0.05; Fig. S11a) and ION (− 0.932, *P* < 0.005; Fig. S11b); the total N-terminus and C-terminus immunolabelling also exhibited a strong negative correlation in the ION (− 0.841, *P* < 0.05; Fig. S11c).

## Discussion

Over the past decade, a growing body of literature has emerged that suggests that the constituent α-Syn structures that potentiate the formation and growth of pathogenic aggregates in PD and MSA exhibit disease-specific biochemical and ultrastructural differences [1, 2, 47–49]. The structural heterogeneity of α-Syn – both within and between α-synucleinopathies – can be partially explained by its susceptibility to post-translational modification. In addition to phosphorylation, ubiquitination, and nitration, several disease-associated truncational variants of α-Syn also exist; however, the relative abundance of these truncational variants in PD, and especially MSA, remains poorly characterised. In addition to post-translational modifications, α-Syn exhibits extensive microenvironment-dependent conformational variability within the human brain. Given the structural diversity of α-Syn and its differential conformational malleability within the complex milieu of human α-synucleinopathies, it stands to reason that the abundance and exposure of different α-Syn post-translational modifications and epitopes would also differ across diseases, necessitating the use of different α-Syn antibodies.

Our recent identification of α-Syn conformers in human brains with PD that were exclusively N-terminus-immunoreactive [13] prompted the subsequent investigation of differential epitope-specific immunolabelling in human brains with MSA. In the present study, using our previously described panel of α-Syn antibodies, we showed that the N-terminus immunolabelling consistently identified the highest proportion of α-Syn pathology across aggregate-prone regions of both MSA and PD. We further demonstrated that the pS129 immunolabelling detected significantly less α-Syn pathology in MSA, compared to N-terminus and C-terminus immunolabelling. Finally, we demonstrated that the C-terminus immunolabelling was significantly higher in MSA compared to PD.

Whilst the total N-terminus immunolabelling was relatively comparable between PD and MSA, the unique epitope-specific N-terminus immunolabelling was consistently higher in PD than in MSA. This can partially be explained by the greater proportion and diversity of exclusively N-terminus-immunoreactive α-Syn conformers in PD. To this end, while perinuclear, microglial, and astrocytic α-Syn aggregates were abundant in PD, we did not observe these unique N-terminus α-Syn conformers in MSA. We did, however, observe the presence of punctate intracellular inclusions within the neuronal cytoplasm across all MSA regions, albeit to a much lesser extent than was observed in PD. These exhibited an identical morphology to the neuronal lysosomal aggregates we have previously observed in PD [13]. In addition, we identified a previously uncharacterised population of neuronal cytoplasmic and intranuclear inclusions within the ION that exhibited exclusive N-terminus immunoreactivity. While neuronal inclusions have previously been reported within the ION [27, 31], these studies have utilised single-stain methodologies with C-terminus α-Syn antibodies. These reservoirs of neuronal inclusion pathology within the ION could potentially manifest as a primary source of α-Syn seeds that are preferentially taken up by oligodendrocytes, establishing a pathological nidus within the oligodendroglial cellular milieu. The relatively high level of unique epitope-specific N-terminus immunolabelling in the ION and indeed all interrogated regions, highlights the substantial proportion of α-Syn pathology that cannot be captured by pS129 and other canonical C-terminus antibodies. Future investigations are needed to elucidate whether these unique N-terminus neuronal inclusions represent C-terminally-truncated variants of α-Syn, or disease- and region-specific conformational variants, within which the target C-terminus epitopes are masked. Importantly, the diversity and true abundance of neuronal inclusions revealed by N-terminus immunolabelling – which otherwise would go undetected with traditional solitary pS129 immunolabelling – could have substantial implications for studies that correlate levels of neuronal loss to local neuronal inclusion pathology [26].

The most striking finding of the present study was that pS129 immunolabelling failed to capture the bulk of α-Syn pathology across all studied regions of both PD and MSA brains. To this end, even in the PD hippocampus, the region with the highest proportion of pS129 immunolabelling, only 41.91% ± 7.55% of the total α-Syn pathology was detected by the pS129 antibody. Across all regions, pS129 immunolabelling only detected 28.76% ± 20.35% of the total α-Syn pathology in PD, and 21.82% ± 16.61% of the total α-Syn pathology in MSA, which means that pS129 immunolabelling failed to capture over 70% of the true α-Syn pathology in both MSA and PD. Our findings support those of Peng et al*.* (2018), who reported—using the same pS129 α-Syn antibody (81A)—that the extent of S129 phosphorylation in α-Syn fibrils extracted from patients with MSA was significantly less than in α-Syn fibrils extracted from patients with Lewy body diseases [47]. It is important to note that 81A, the pS129 antibody used in the present study, is insensitive to neighbouring post-translational modifications, unlike other commonly used pS129 antibodies, and detects pS129 α-Syn that is also phosphorylated at Y125, or truncated at residue 133 or 135 [50]. As part of our initial validations, we compared the immunoreactivity of 81A with that of EP1536Y, another commonly used pS129 antibody. We found comparable immunolabelling of both pS129 antibodies under a range of different antigen retrieval conditions (Fig. S12). For these reasons, combined with the added flexibility afforded by its isotype subtype-specificity in the way of multiplex panel design, we chose to use the 81A pS129 antibody in the present study.

The C-terminus antibody used in this study (MJFR1) targets residues 118–123, which encompasses the Asp-119 and Asn-122 residues, two of the most common cleavage sites of α-Syn [35]. Interestingly, C-terminus immunolabelling detected a significantly higher proportion of α-Syn in MSA (65.95% ± 16.98%) than in PD (27.79% ± 20.04%). Given the location of these common truncation sites, this differential C-terminus immunoreactivity could suggest that there is a higher proportion of C-terminally-truncated α-Syn in PD relative to MSA. Although several pathologically significant truncation sites have been identified in PD [35, 37, 51–54], very few studies have investigated the extent of α-Syn truncation in MSA. Interestingly, Hass et al. (2021) reported a relatively high abundance of α-Syn truncated at residue 125 in the cerebellum and pons of MSA brains [37]. Coupled with our identification of neuronal α-Syn inclusions within the ION that exhibited exclusive N-terminus immunoreactivity and the fact that 34.05% of MSA pathology was not detected by MJFR1, these data suggest that additional C-terminally truncated variants – cleaved after residue 123 – could be present in the human brain with MSA. Alternatively, this differential C-terminus immunoreactivity between PD and MSA could be explained by the disease-specific conformation-induced masking of C-terminus epitopes. Future investigations using α-Syn antibodies that detect additional epitope sequences across the C-terminus domain would help further interrogate both these hypotheses. Moreover, additional validations of potential late C-terminus truncations would provide further clarity around the reason for lower pS129 immunoreactivity in MSA. Unfortunately, from our data alone, it is unclear whether this decreased pS129 immunoreactivity is due to a genuine lack of S129 phosphorylation in MSA, or due to the proteolytic cleavage of S129 between residues 124–128.

**Fig. 6:**
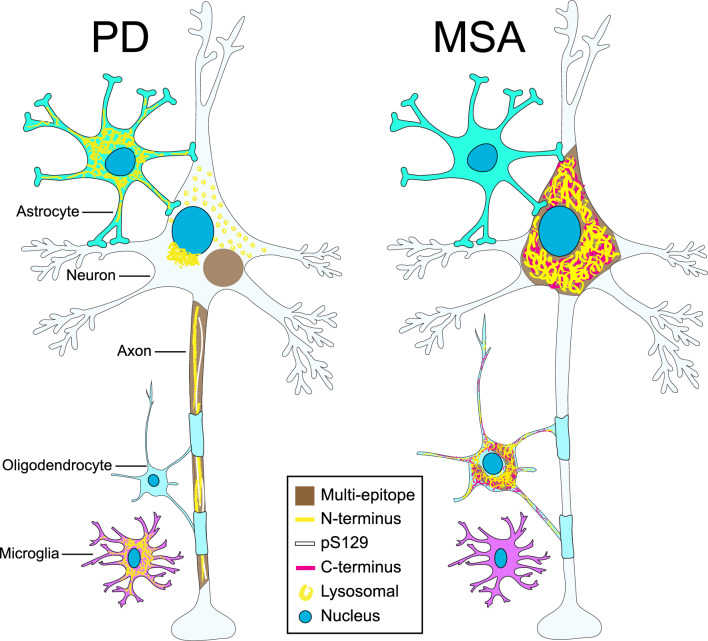
Illustration summarising the prevailing α-Syn aggregate structures that are present in PD and MSA. In the human brain with PD, novel perinuclear, microglial, astrocytic, and lysosomal α-Syn aggregate structures that exhibit epitope-specific N-terminus (residues 34–57) immunoreactivity were observed. In the human brain with MSA, microglial and astrocytic inclusions were not observed. Punctate neuronal inclusions with exclusive N-terminus immunoreactivity were often observed; however, these were considerably less frequent than in PD (not included in the illustration). Structurally discrete, whisp-like α-Syn filaments with either exclusive N-terminus or exclusive C-terminus immunoreactivity were frequently observed, which appeared to be interlaced and juxtaposed with one another. Finally, in the MSA ION, several neuronal inclusions with exclusive N-terminus immunoreactivity were observed (not included in the illustration)

In contrast to PD, where mature α-Syn aggregates typically exhibited a condensed and structurally well-defined Lewy body or Lewy neurite morphology, inclusions in MSA were composed of discrete interlacing α-Syn filaments, the boundaries of which were structurally circumscribed by the intracellular space. Strikingly, these discrete α-Syn filaments often exhibited exclusive N-terminus or exclusive C-terminus immunoreactivity. Further investigations are currently underway to elucidate the biological and potentially conformational relevance of this epitope-specific distribution among discrete inclusion filaments. Figure 6 summarises the major epitope-specific α-Syn proteoforms present in PD and MSA.

## Conclusion

Collectively, our findings highlight the remarkable deficit of pS129 to accurately capture the diversity of α-Syn variants within both PD and MSA. Our study challenges traditional tenets of pS129 as a putative hallmark of pathological α-Syn and is of particular relevance, given that pS129 α-Syn antibodies are so heavily relied on in both research and pathology laboratories worldwide [50]. Notwithstanding the implications of this incomplete capture for biological research, these findings are especially pertinent in the context of pathology, where single-stain methodologies are commonplace. Based on our present and previous findings [13], we propose that antibodies targeting the N-terminus (namely residues 34–57) of α-Syn should be used routinely in all pathology and research labs. Detection of these epitopes will ensure that more accurate and meaningful conclusions can be drawn from immunohistochemical interrogations of human α-synucleinopathies. Where possible, however, we maintain that utilising a multiplex immunohistochemical approach to label multiple α-Syn epitopes simultaneously is critical to ensure the precise and complete capture of this structurally diverse protein within the complex milieu of human α-synucleinopathies.

## Supplementary Information

Additional file 1. **Supplementary Methods.** **Table S1**. Case information for human brain tissue used in this study. **Table S2**. Primary antibodies used for immunohistochemistry. **Table S3**. Secondary antibodies used for immunohistochemistry. **Table S4**. Mean total pathology load (percentage of area) for each individual α-Syn antibody across PD and MSA regions. **Table S5**. Mean total and unique epitope-specific immunolabelling of each antibody across all regions. **Figure S1**. Schematic diagram summarising the quantification of total α-Syn (all epitopes), total epitope (individual α-Syn epitope), and unique epitope-specific α-Syn. **Figure S2**. Representative overview of the segmentation process utilised in this study. **Figure S3**. Representative confocal images demonstrating the morphological heterogeneity and epitope-specific α-synuclein immunolabelling of α-synuclein inclusions in the medulla, substantia nigra, hippocampus, and cerebellum of MSA-C. **Figure S4**. Representative confocal images demonstrating the morphological heterogeneity and epitope-specific α-synuclein immunolabelling of α-synuclein inclusions in the medulla, substantia nigra, hippocampus, and cerebellum of MSA-P. **Figure S5**. Immunostaining of the N-terminus, pS129, and C-terminus α-Syn antibodies in neurologically normal (substantia nigra) and MSA brain tissue (substantia nigra and medulla). **Figure S6**. Representative single-channel confocal images depicting the immunolabelling profile of N-terminus α-Syn (yellow), pS129 α-Syn (cyan), and C-terminus α-Syn (magenta) in glial cytoplasmic inclusions and neuronal inclusions in the human brain with MSA. **Figure S7**. Representative confocal images with superimposed fluorescent profiles demonstrating epitope-specific α-Syn immunolabelling in neuronal and oligodendroglial inclusions in MSA at different Z-stack levels. **Figure S8**. Morphological demarcation of oligodendroglial and neuronal α-synuclein inclusions by the cellular periphery. **Figure S9**. Distribution of α-Syn pathology (yellow) in the MSA medulla. **Figure S10**. Statistically significant correlations between N-terminus, pS129, and C-terminus (epitope-specific and total) immunolabelling in PD. **Figure S11**. Statistically significant correlations between N-terminus, pS129, and C-terminus (epitope-specific and total) immunolabelling in MSA. **Figure S12**. Representative single channel and merged confocal images depicting the comparable immunolabelling profile of the ab184674 (81A) and ab51253 (EP1536Y) pS129 α-Syn antibodies in both PD and MSA.

Additional file 2. Profile Video S1.

Additional file 3. Profile Video S2.

Additional file 4. Profile Video S3.

Additional file 5. Profile Video S4.

Additional file 6. Profile Video S5.

Additional file 7. Video S1

Additional file 8. Video S2

Additional file 9. Video S3

Additional file 10. Video S4

Additional file 11. Video S5

Additional file 12. Video S6

Additional file 13. Video S7

## Data Availability

All data are available in the main text or Supplementary Materials. The raw data that support the findings of this study are available from the corresponding author upon reasonable request.

## Abbreviations

α-Syn: Alpha-synuclein
ABC: Alzheimer’s Disease Neuropathologic Change Scoring
ANOVA: Analysis of variance
GCIs: Glial cytoplasmic inclusions
ION: Inferior olivary nucleus
LP: Lewy pathology
LSM: Laser scanning microscopy
MAP2: Microtubule-associated protein 2
MSA: Multiple system atrophy
NAC: Non-amyloid component
NA: Numerical aperture
NCI: Neuronal cytoplasmic inclusions
OPCA: Olivopontocerebellar atrophy
PD: Parkinson’s disease
PMD: Post-mortem delay
PMT: Photomultiplier tube
pS129: Phosphorylated serine 129
PTM: Post-translational modification
SD: Standard deviation
SND: Striatonigral degeneration
STED: Stimulated emission depletion
TPPP: Tubulin polymerization-promoting protein
