# Supplementary material for: N-terminus α-synuclein detection reveals new and more diverse aggregate morphologies in multiple system atrophy and Parkinson’s disease
Source: Transl Neurodegener. 2024 Dec 27;13:67. doi: 10.1186/s40035-024-00456-3 (PMC11673343; doi:10.1186/s40035-024-00456-3)
Supplement: Supplementary file 1 — Additional file 1. Supplementary Methods. Table S1. Case information for human brain tissue used in this study. Table S2. Primary antibodies used for immunohistochemistry. Table S3. Secondary antibodies used for immunohistochemistry. Table S4. Mean total pathology load (percentage of area) for each individual α-Syn antibody across PD and MSA regions. Table S5. Mean total and unique epitope-specific immunolabelling of each antibody across all regions. Figure S1. Schematic diagram summarising the quantification of total α-Syn (all epitopes), total epitope (individual α-Syn epitope), and unique epitope-specific α-Syn. Figure S2. Representative overview of the segmentation process utilised in this study. Figure S3. Representative confocal images demonstrating the morphological heterogeneity and epitope-specific α-synuclein immunolabelling of α-synuclein inclusions in the medulla, substantia nigra, hippocampus, and cerebellum of MSA-C. Figure S4. Representative confocal images demonstrating the morphological heterogeneity and epitope-specific α-synuclein immunolabelling of α-synuclein inclusions in the medulla, substantia nigra, hippocampus, and cerebellum of MSA-P. Figure S5. Immunostaining of the N-terminus, pS129, and C-terminus α-Syn antibodies in neurologically normal (substantia nigra) and MSA brain tissue (substantia nigra and medulla). Figure S6. Representative single-channel confocal images depicting the immunolabelling profile of N-terminus α-Syn (yellow), pS129 α-Syn (cyan), and C-terminus α-Syn (magenta) in glial cytoplasmic inclusions and neuronal inclusions in the human brain with MSA. Figure S7. Representative confocal images with superimposed fluorescent profiles demonstrating epitope-specific α-Syn immunolabelling in neuronal and oligodendroglial inclusions in MSA at different Z-stack levels. Figure S8. Morphological demarcation of oligodendroglial and neuronal α-synuclein inclusions by the cellular periphery. Figure S9. Distribution of α-Syn pathology ( [file 40035_2024_456_MOESM1_ESM.pdf]

Supplementary Materials

N-terminus α-synuclein detection reveals new and more diverse aggregate morphologies in multiple system atrophy and Parkinson's disease

James A Wiseman[1,2,3], YuHong Fu[3], Richard L M Faull[1,2], Clinton P Turner[4], Maurice A Curtis[1,2], Glenda M Halliday[3,5], Birger V Dieriks[1,2,3].

1 Department of Anatomy and Medical Imaging, University of Auckland, Auckland, New Zealand

2 Centre for Brain Research, University of Auckland, Auckland, 1023, New Zealand

3 Brain and Mind Centre & Faculty of Medicine and Health School of Medical Sciences, The University of Sydney, Sydney, NSW 2050, Australia

4 LabPlus, Department of Anatomical Pathology, Te Whatu Ora, Auckland, New Zealand

5 Neuroscience Research Australia & Faculty of Medicine School of Medical Sciences, University of New South Wales, Sydney, NSW 2052, Australia

Correspondence to:

Birger Victor Dieriks

Department of Anatomy and Medical Imaging

85 Park Road, Grafton

Auckland 1142

v.dieriks@auckland.ac.nz

1 Supplementary Methods

1.1 Cohorts and tissue preparation

Human post-mortem brain tissue used in this study was received from (1) the Sydney Brain Bank, and (2) the Neurological Foundation Human Brain Bank (New Zealand). All brain tissue was donated with written informed consent from donors and their families prior to brain removal and all protocols were approved by the University of Sydney Human Research Ethics Committee (2019/491) and the University of Auckland Human Participants Ethics Committee (Ref: 011654). All experiments were conducted in accordance with relevant guidelines and regulations. All cases used in this study were assessed by a neuropathologist. All MSA cases (n = 10) had a clinical history of MSA-P (n = 5) or MSA-C (n = 5), and pathological features were consistent with MSA pathology as confirmed by a neuropathologist. Key neuropathological features were the presence of α-synuclein immunopositive neuronal and glial cytoplasmic inclusions in the medulla, inferior olivary nuclei, nucleus of the solitary tract, substantia nigra, hippocampus and cerebellum. MSA cases had a disease duration ranging from 4–22 years, with the mean duration being 10.1 ± 5.1 years (Table 1). The mean age of MSA cases was 67.7 ± 8.5 and ranged from 54–84 years; the mean post-mortem delay was 24.6 ± 11.6 hours with a range of 7–45 hours (Table 1). All PD cases (n = 10) had a clinical history of PD, and pathological features were consistent with PD pathology as confirmed by a neuropathologist. Key neuropathological features were loss of pigment and pigmented cells in the substantia nigra and accumulation of LBs in the substantia nigra and other brain regions; many cases also had evidence of cortical LB disease. PD cases had a disease duration ranging from 9–26 years, with the mean duration being 17.1 ± 6.6 (Table 1). The mean age of PD cases was 74.9 ± 5.3 and ranged from 65–80 years; the mean post-mortem delay was 12.3 ± 7.1 hours with a range of 2.25–25 hours (Table 1). The neurologically normal cases (n = 5) had no clinical history of neurological abnormalities, and no other significant neuropathology was noted upon post-mortem examination. The mean age (± SD) of normal cases was 81.8 ± 11.6 and ranged from 63–93 years (Table 1). The mean post-mortem delay of normal cases was 26.2 ± 7 hours with a range of 19–36 hours.

1.2 Formalin-fixed paraffin-embedded tissue processing

Upon receipt of the brain, the right hemisphere of each brain was fixed by perfusion of 15% formaldehyde in 0.1 M phosphate buffer through the cerebral arteries and subsequently dissected into anatomically significant blocks as previously described[1,2]. A 5 mm-thick section was sampled from each block for paraffin embedding and the remaining tissue was snap-frozen using powdered dry ice and stored at -80°C. All olfactory bulbs were removed from the brain prior to perfusion to preserve

olfactory tissue integrity. Olfactory bulbs were subsequently immersion fixed in 15% formaldehyde in 0.1 M phosphate buffer for 24 hours at room temperature. Both the brain tissue blocks and olfactory bulbs were processed for paraffin embedding as previously described[3]. Paraffin blocks were sequentially sectioned using a rotary microtome (Leica Biosystems, RM2335) at a thickness of 7 µm. Olfactory bulb and cerebellar blocks were sectioned in the sagittal plane, medulla oblongata and substantia nigra blocks were sectioned in the horizontal plane, and hippocampal and cortical blocks were sectioned in the coronal plane. Sections were individually mounted onto Über plus charged microscope slides (IntstrumeC) using a 41°C-water bath (Leica Biosystems, H1210). Mounted sections were desiccated at room temperature for 72 hours.

1.3 Paraffin-embedded fluorescent immunohistochemistry

Mounted formalin-fixed paraffin-embedded tissue sections were heated on a 60°C hot plate for 1 hour to melt the embedding paraffin. Paraffin wax was removed by submerging tissue sections in two consecutive 100% xylene baths (2 x 30 minutes). Tissue sections were rehydrated by sequentially immersing slides in a series of ethanol baths (100% EtOH, 2 x 15 minutes; 95%, 80% and 75% EtOH, 1 x 10 minutes) followed by 3 x 5 minutes washes in ddH_2O. Heat-induced epitope retrieval was performed by heating slides in a Tris-EDTA (pH 9.0, 0.05% Tween 20) buffer (Abcam) in a pressure cooker (2100 Antigen Retriever, Aptum Biologics Ltd.) at 121°C for 20 minutes. Sections were cooled for 1.5 hours and washed in ddH_2O (3 x 5 minutes). Secondary acid-induced epitope retrieval was performed by incubating sections for 4 minutes in 99% formic acid, after which sections were washed in ddH_2O (3 x 5 minutes). Hydrophobic wax barriers were drawn around tissue sections using an ImmEdge Hydrophobic Barrier PAP pen (Vector Laboratories) and sections were permeabilised for 15 minutes in 4°C PBS-T (PBS with 0.2% Triton™ X-100; Sigma-Aldrich, T9284). Tissue sections were washed (3 x 5 minutes in PBS) and incubated for 1 hour in 10% normal goat serum (in PBS) to prevent non-specific secondary antibody binding (ThermoFisher, 16210-072). Sections were incubated with primary antibodies diluted in 1% normal goat serum overnight at 4°C in a humidified slide chamber (Supplementary Table 2). Sections were subsequently washed in PBS (3 x 5 minutes). Secondary antibodies, diluted in 1% normal goat serum with Hoechst 33342 nuclear counterstain (1:20,000; ThermoFisher, H1399), were incubated on sections for 3 hours at room temperature (Supplementary Table 3). Slides were washed in PBS (3 x 5 minutes) and coverslipped (Menzel-Gläser; #1.5) using Prolong® Diamond Antifade Mountant (ThermoFisher). Coverslips were sealed with nail polish and slides were stored at 4°C, protected from light. All immunohistochemical interrogations in this study included validations with no-primary controls, in which primary antibodies were not added. These validations were conducted to control for background autofluorescence signatures and any non-specific

antibody labelling. Any autofluorescence signature fell below the segmentation threshold of interrogated aggregate morphologies.

To ensure that the observed epitope-specific immunolabelling was not the result of steric hindrance caused by competing differential antibody-epitope affinities, a series of progressive single-label antibody validations were conducted, as previously described[4]. No detectable differences in epitope-specific immunoreactivities were observed between the simultaneously immunolabelled sections and the serially immunolabelled sections, thereby mitigating steric hindrance as a confounding influence.

2 Supplementary Tables

Table S1. Case information for human brain tissue used in this study.

Case	Neuropathological Diagnosis	Age	Sex	PMD (hours)	Cause of Death	Duration with Disease (Years)
MSA 1	MSA-C	66	F	15	Cardiorespiratory failure	7
MSA 2	MSA-C	69	M	41	Aspiration pneumonia	14
MSA 3	MSA-C	70	M	45	Pneumonia	7
MSA 4	MSA-C	54	M	27	Cardiorespiratory failure	10
MSA 5	MSA-C	74	F	20	Renal failure	7
MSA 6	MSA-P	61	M	21	Aspiration pneumonia	12
MSA 7	MSA-P	61	M	7	Respiratory arrest	4
MSA 8	MSA-P	84	F	30	Cardiorespiratory failure	22
MSA 9	MSA-P	74	F	18	Sepsis	8
MSA 10	MSA-P	64	M	22	Bronchopneumonia	10
PD 1	PD	79	F	25	Renal failure	9
PD 2	PD	78	M	6	Aspiration pneumonia	19
PD 3	PD	74	M	10.5	End stage PD and DLB	12
PD 4	PD	80	F	14	Bronchopneumonia	–
PD 5	PD	80	M	18	Urosepsis	26
PD 6	PD	67	M	2.25	End stage PD and DLB	9
PD 7	PD	73	M	17.5	Aspiration pneumonia	22
PD 8	PD	65	M	17	Pneumonia	12
PD 9	PD	76	F	6.5	Abdominal carcinoma	23
PD 10	PD	77	M	6.5	End stage Lewy body disease	22
Control 1	Neurologically normal	84	M	36	Pulmonary hypertension	N/a
Control 2	Neurologically normal	80	F	29	Cardiac failure	N/a
Control 3	Neurologically normal	89	M	27	Pulmonary embolism	N/a
Control 4	Neurologically normal	63	M	20	Asphyxia	N/a
Control 5	Neurologically normal	93	F	19	Pneumonia	N/a

Table S2. Primary antibodies used for immunohistochemistry.

Antibody	Epitope	Species	Isotype	Manufacturer	Immunogenic Labelling	Catalogue No.	Dilution
α-synuclein A15110D	34-45	Mouse	Monoclonal IgG1	BioLegend, San Diego, California, United States	N-terminus	849102	1:1000
α-synuclein MJFR1	118-123	Rabbit	Monoclonal IgG	Abcam, Cambridge, United Kingdom	C-terminus	Ab138501	1:1000
α-synuclein (81A)	pS129	Mouse	Monoclonal IgG2a	Abcam, Cambridge, United Kingdom	Phosphorylated serine 129	Ab184674	1:4000
α-synuclein (EP1536Y)	pS129	Rabbit	Monoclonal IgG	Abcam, Cambridge, United Kingdom	Phosphorylated serine 129	Ab51253	1:4000
p25α/TPPP	–	Goat	Polyclonal	ThermoFisher Scientific	Oligodendrocyte marker/p25α	PA5-19243	1:500
NeuN	–	Guinea Pig	Polyclonal	MilliporeSigm, Burlington, Massachusetts, United States	Neuronal nuclei marker	ABN90	1:1000
Lamin	–	Rabbit	Polyclonal IgG	Abcam, Cambridge, United Kingdom	Nuclear envelope	Ab108595	1:500
MAP2	–	Chicken	Polyclonal IgY	Antibodies.com, Cambridge, United Kingdom	Neuronal cell marker	A85363	1:500
LAMP1	–	Mouse	Monoclonal IgG2a	Santa Cruz, Dallas, Texas, United States	Lysosomal marker	sc-17768	1:100
MBP	–	Rabbit	Polyclonal	MilliporeSigm, Burlington, Massachusetts, United States	Myelin basic protein	HPA 049222	1:1000

*N-terminus α-Syn antibody does not cross-react with β-synuclein and γ-Synuclein antibodies [13].

Table S3. Secondary antibodies used for immunohistochemistry.

Antibody	Target	Species	Isotype	Manufacturer	Catalogue No.	Dilution
Goat anti-mouse AlexaFluor 488	Mouse	Goat	IgG (H+L)	ThermoFisher, Waltham, Massachusetts, United States	A11001	1:500
Goat anti-mouse AlexaFluor 488	Mouse	Goat	IgG1	ThermoFisher, Waltham, Massachusetts, United States	A21121	1:500
Goat anti-mouse AlexaFluor 488	Mouse	Goat	IgG2a	ThermoFisher, Waltham, Massachusetts, United States	A21131	1:500
Goat anti-rabbit AlexaFluor 488	Rabbit	Goat	IgG (H+L)	ThermoFisher, Waltham, Massachusetts, United States	A11034	1:500
Goat anti-chicken AlexaFluor 488	Chicken	Goat	IgG (H+L)	ThermoFisher, Waltham, Massachusetts, United States	A11039	1:500
Goat anti-mouse AlexaFluor 594	Mouse	Goat	IgG (H+L)	ThermoFisher, Waltham, Massachusetts, United States	A11032	1:500
Goat anti-mouse AlexaFluor 594	Mouse	Goat	IgG1	ThermoFisher, Waltham, Massachusetts, United States	A21125	1:500
Goat anti-mouse AlexaFluor 594	Mouse	Goat	IgG2a	ThermoFisher, Waltham, Massachusetts, United States	A21135	1:500
Goat anti-rabbit AlexaFluor 594	Rabbit	Goat	IgG (H+L)	ThermoFisher, Waltham, Massachusetts, United States	A11037	1:500
Goat anti-chicken AlexaFluor 594	Chicken	Goat	IgG (H+L)	ThermoFisher, Waltham, Massachusetts, United States	A11042	1:500
Goat anti-mouse AlexaFluor 647	Mouse	Goat	IgG2c	Jackson ImmunoResearch, West Grove, Pennsylvania, United States	115-607-188	1:500
Goat anti-guinea pig AlexaFluor 647	Guinea Pig	Goat	IgG (H+L)	ThermoFisher, Waltham, Massachusetts, United States	A21450	1:500
Goat anti-rabbit AlexaFluor 647	Rabbit	Goat	IgG (H+L)	ThermoFisher, Waltham, Massachusetts, United States	A21245	1:500
Goat anti-chicken AlexaFluor 647	Chicken	Goat	IgG (H+L)	ThermoFisher, Waltham, Massachusetts, United States	A21449	1:500
Goat anti-rabbit AlexaFluor 800	Rabbit	Goat	IgG (H+L)	LI-COR, Lincoln, Nebraska, United States	926-32211	1:500
Donkey anti-goat AlexaFluor 647	Goat	Donkey	IgG (H+L)	ThermoFisher, Waltham, Massachusetts, United States	A21447	1:500

Table S4. Mean total pathology load (percentage of area) for each individual α-Syn antibody across PD and MSA regions. MM, middle medulla; SN, substantia nigra; HP, hippocampus.

	N-terminus	pS129	C-terminus
PD MM	0.22	0.03	0.10
PD SN	0.38	0.16	0.20
PD HP	0.52	0.27	0.13
MSA MM	1.78	0.43	1.68
MSA SN	0.81	0.11	0.64
MSA HP	0.14	0.09	0.08

Table S5. Mean total and unique epitope-specific immunolabelling of each antibody across all regions.

	Total			Unique epitope-specific		
**Disease**	**N-terminus**	**pS129**	**C-terminus**	**N-terminus**	**pS129**	**C-terminus**
PD	82.26 ± 12.87%	28.76 ± 20.35%	27.79 ± 20.04%	57.73 ± 20.50%	8.36 ± 9.19%	6.56 ± 7.50%
MSA	77.11 ± 12.23%	21.82 ± 16.61%	65.95 ± 16.98%	26.78 ± 12.00%	5.36 ± 9.22%	16.33 ± 8.28%

3 Supplementary Figures

Total α-synuclein (all epitopes)
Total α-Syn immunolabelling area was defined as the total combined α-Syn immunolabelling area of all α-Syn antibodies. The total α-Syn immunolabelling area was determined by calculating the net α-Syn immunolabelling across the N-terminus (849102), pS129 (Ab184674), and C-terminus (Ab138501) antibodies (both overlapping and exclusive immunolabelling).

Total Epitope (individual epitope)
Total epitope immunoalabelling was defined as the total immunolabelling area for an individual α-Syn antibody (including overlapping area with other α-Syn antibodies). Calculated as a percentage of the total α-Syn immunolabelling area.

Unique Epitope (epitope-specific)
Unique epitope-specific immunolabelling was defined as pathological α-Syn that was exclusively detected by a single α-Syn antibody (excluding overlapping area with other α-Syn antibodies). Calculated as a percentage of the total α-Syn immunolabelling area.

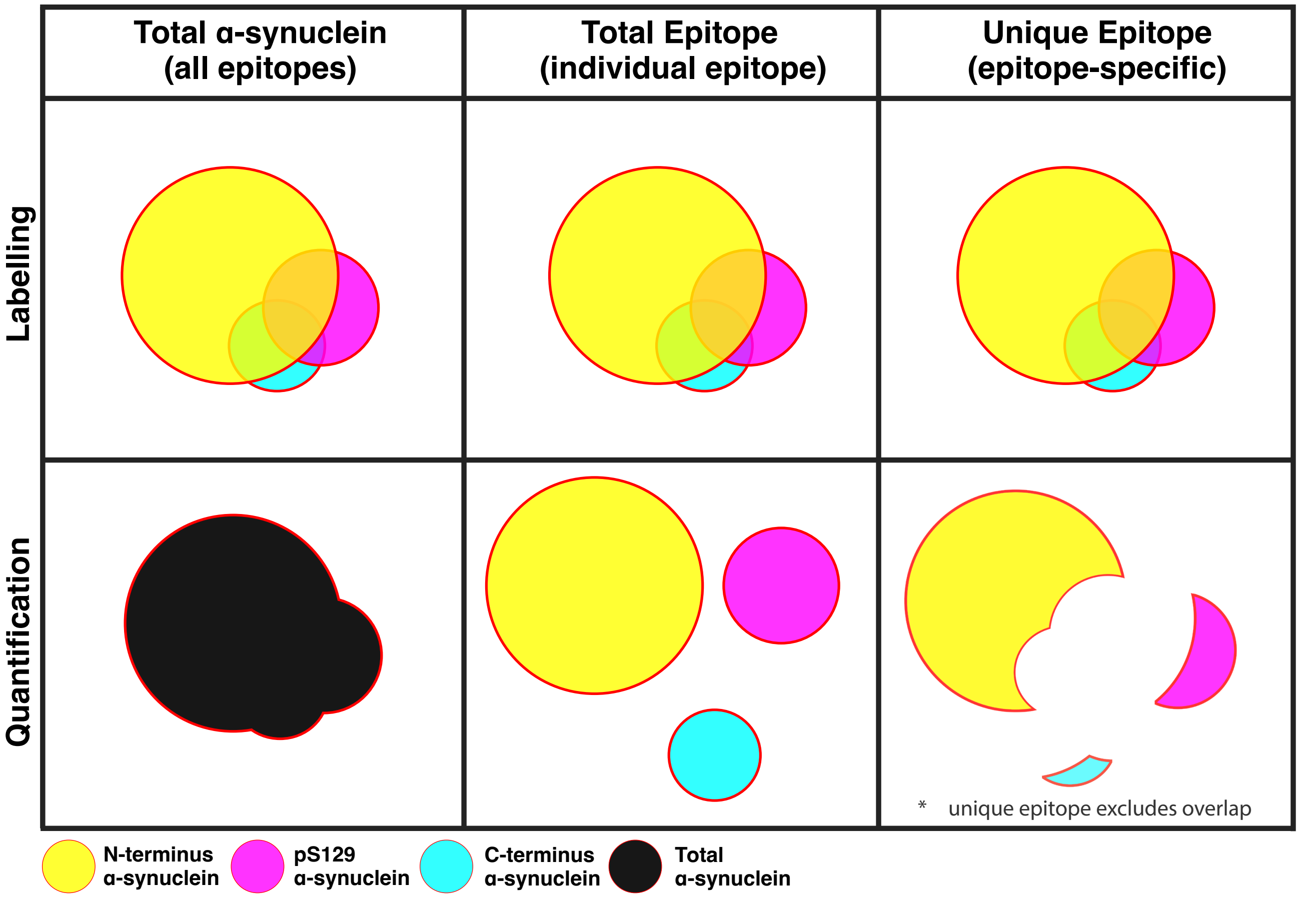

Fig. S1 Schematic diagram summarising the quantification of total α-Syn (all epitopes), total epitope (individual α-Syn epitope), and unique epitope-specific α-Syn.

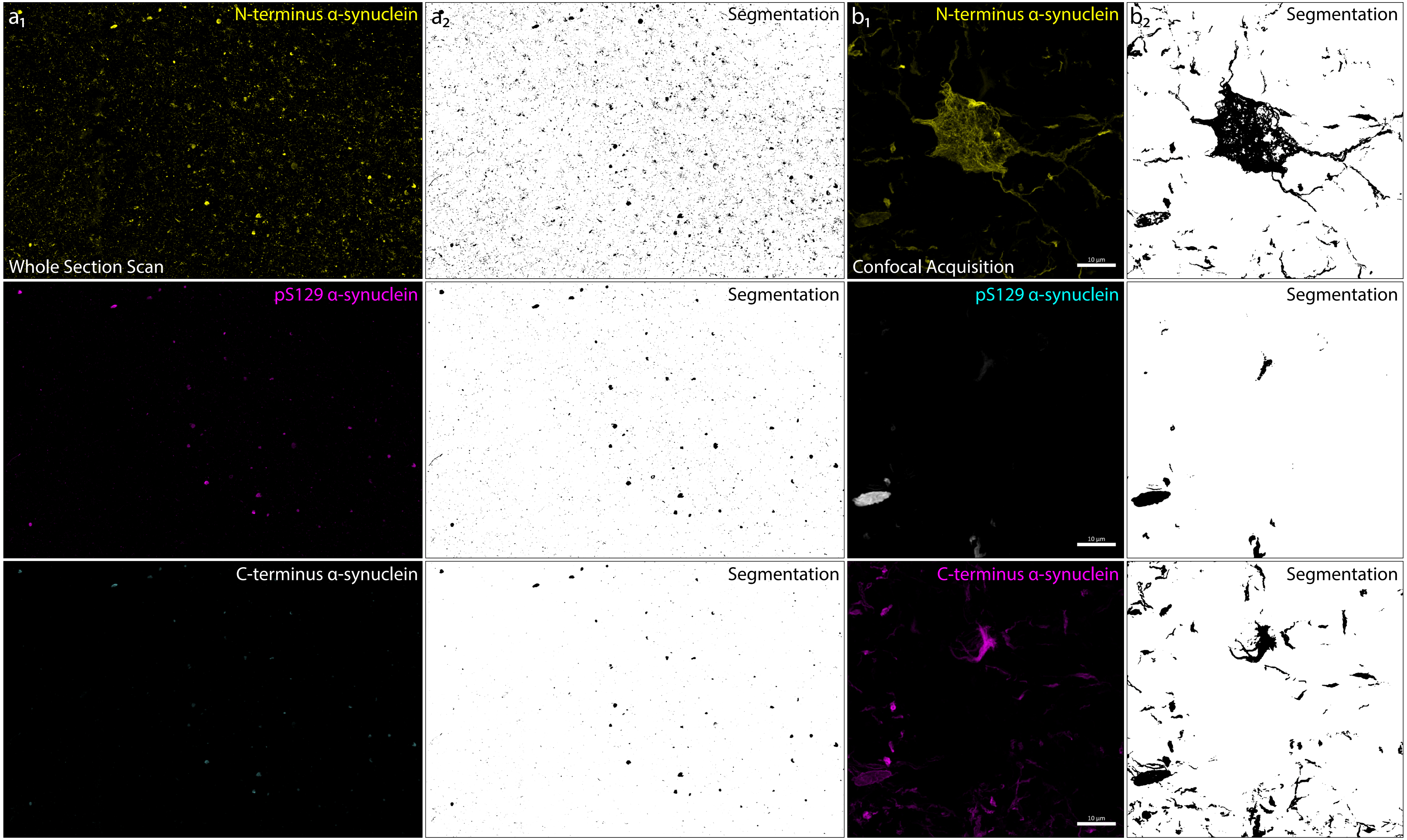

Fig. S2 Representative overview of the segmentation process utilised in this study. (a_1) Single-channel images from a representative whole-section slide scan displaying the total immunolabelling for each of the N-terminus, pS129, and C-terminus α-Syn antibodies. (a_2) Corresponding segmentation mask for each respective α-Syn epitope. (b_1) Single-channel confocal images from a representative confocal acquisition displaying the total immunolabelling for each of the N-terminus, pS129, and C-terminus α-Syn antibodies. (b_2) Corresponding segmentation mask for each respective α-Syn epitope.

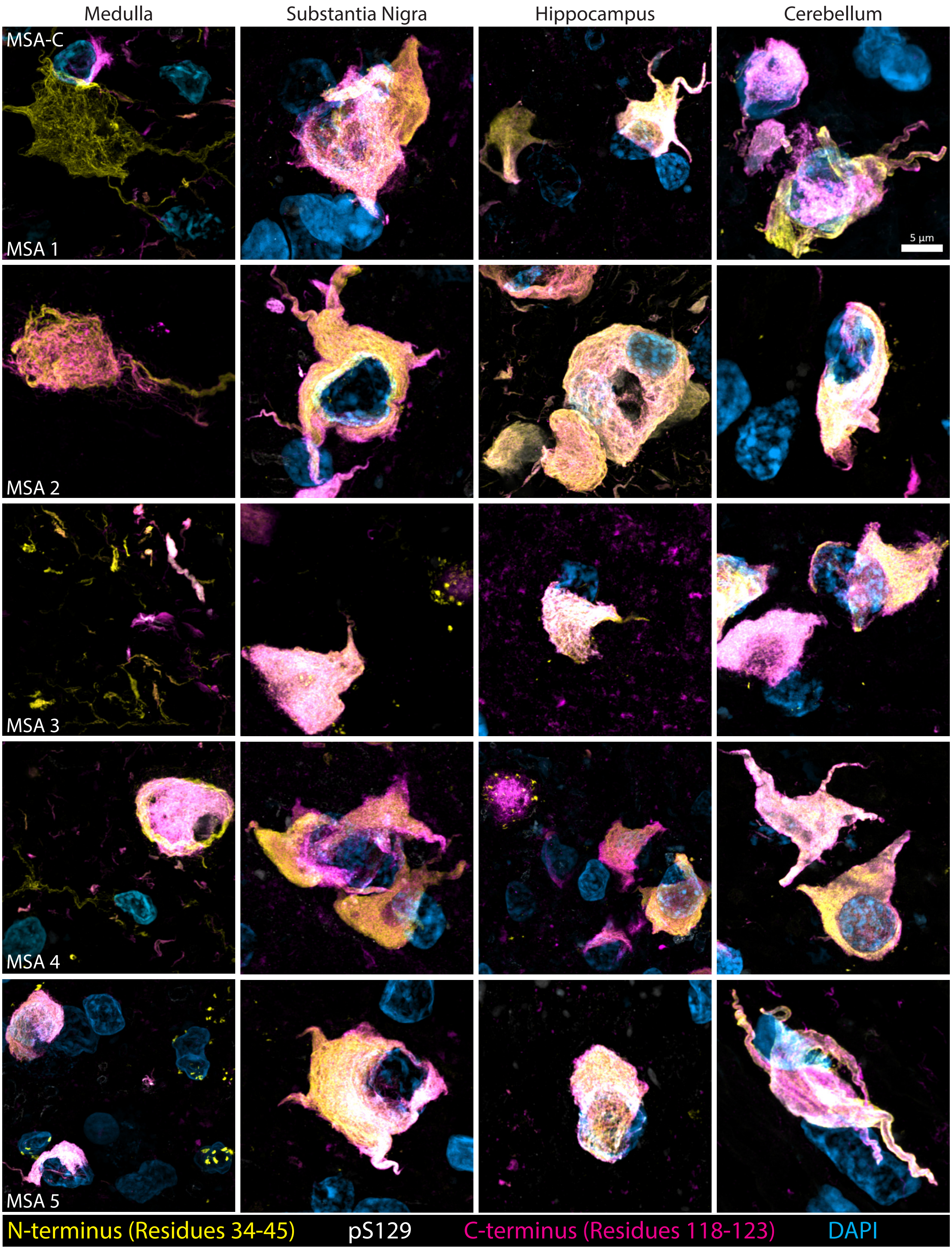

Fig. S3 Representative confocal images demonstrating the morphological heterogeneity and epitope-specific α-synuclein immunolabelling of α-synuclein inclusions in the medulla, substantia nigra, hippocampus, and cerebellum of MSA-C (n = 5). Scale bar, 5 µm.

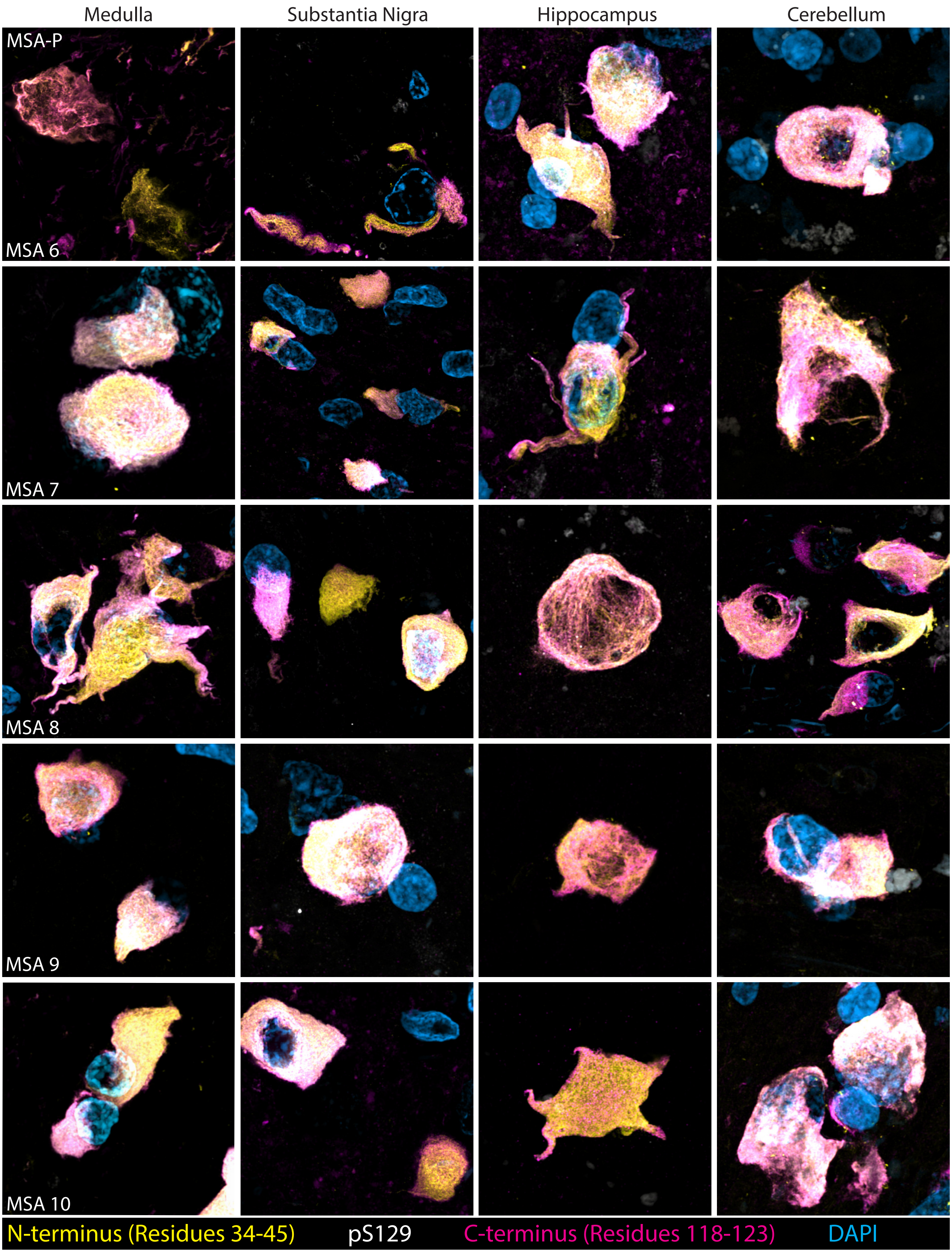

Fig. S4 Representative confocal images demonstrating the morphological heterogeneity and epitope-specific α-synuclein immunolabelling of α-synuclein inclusions in the medulla, substantia nigra, hippocampus, and cerebellum of MSA-P (n = 5). Scale bar, 5 µm.

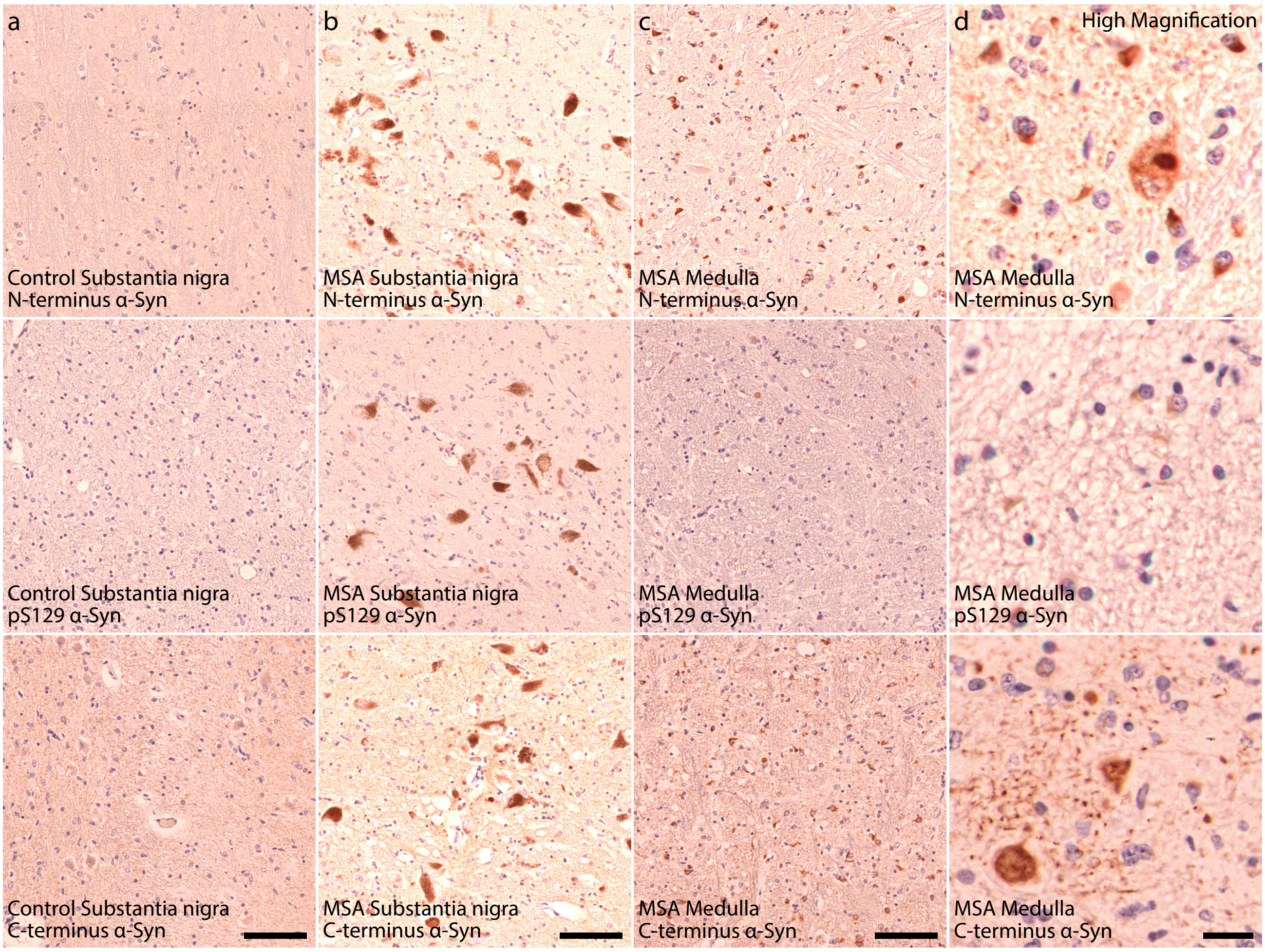

Fig. S5 Immunostaining of the N-terminus, pS129, and C-terminus α-Syn antibodies in neurologically normal (substantia nigra) and MSA brain tissue (substantia nigra and medulla). (a) No pathological α-Syn was observed in neurologically normal cases with any of the α-Syn antibodies. Scale bar, 100 μm. (b) Pathological α-Syn in the MSA substantia nigra and (c) medulla. Scale bar, 100 μm. (d) Higher magnification representative images of pathological α-Syn in the MSA medulla. Scale bar, 20 μm. For all DAB experiments, antibody staining was conducted on sequential sections (within each region), and the same region of interest was acquired during imaging.

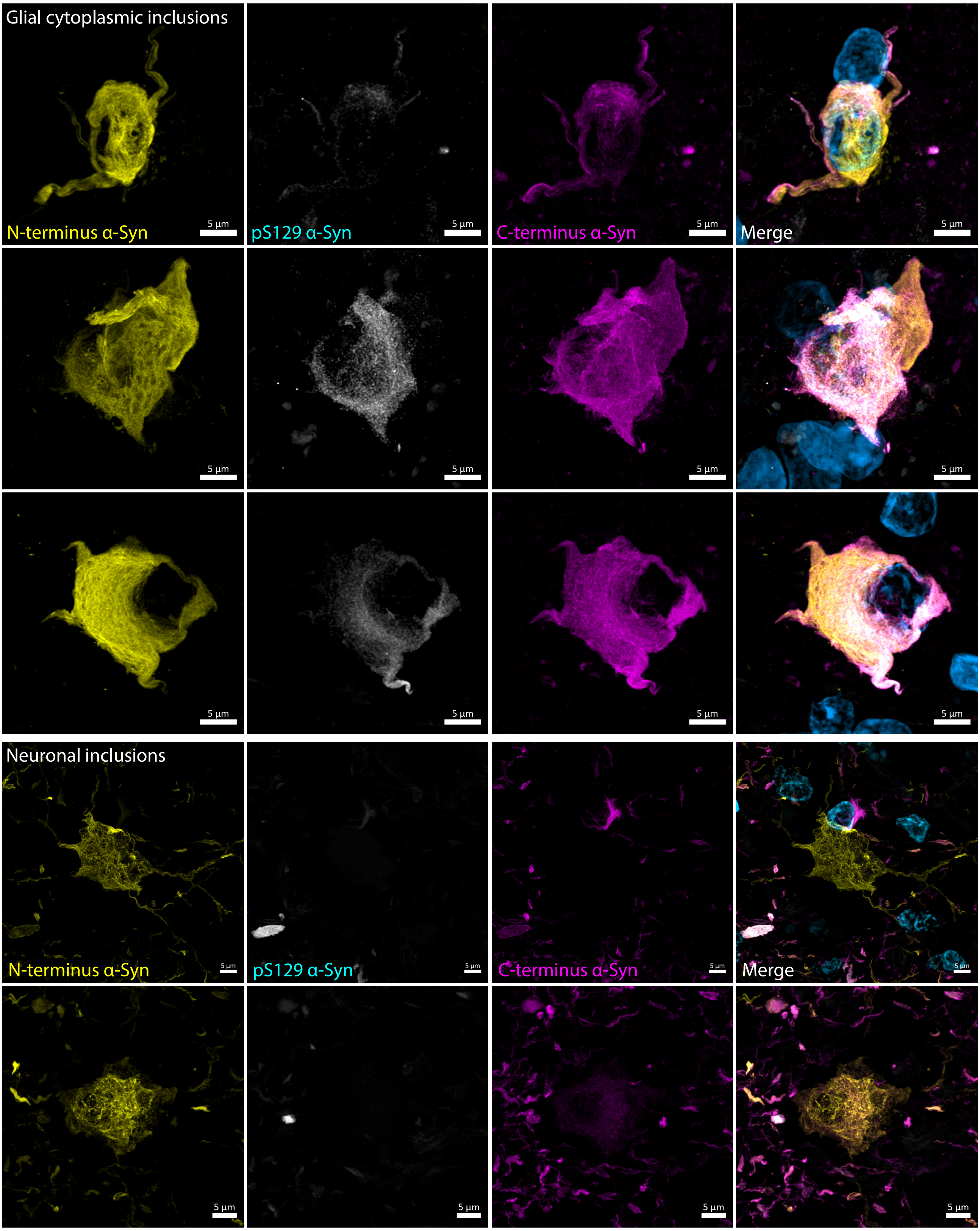

Fig. S6 Representative single-channel confocal images depicting the immunolabelling profile of N-terminus α-Syn (yellow), pS129 α-Syn (cyan), and C-terminus α-Syn (magenta) in glial cytoplasmic inclusions and neuronal inclusions in the human brain with MSA.

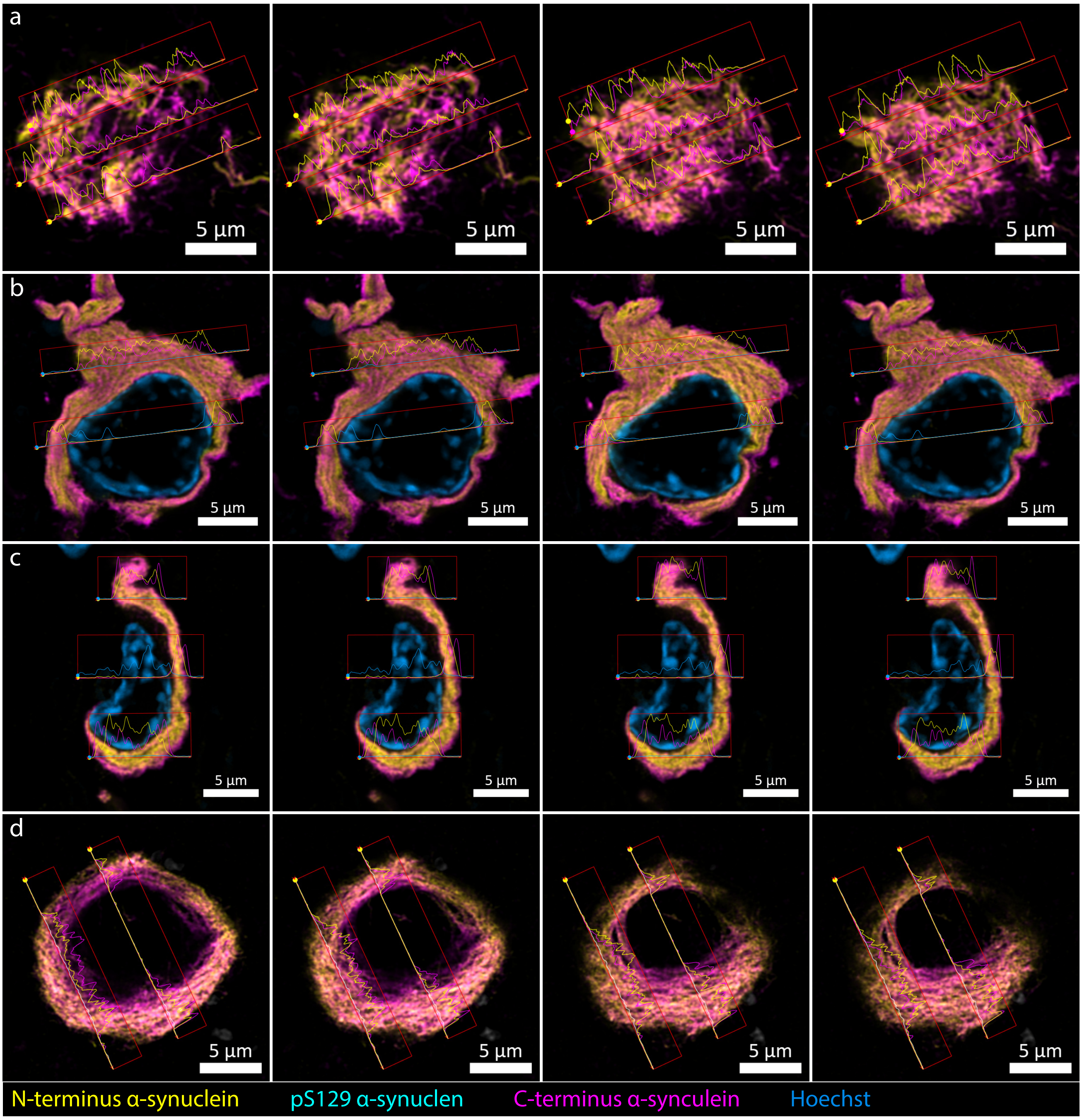

Fig. S7 Representative confocal images with superimposed fluorescent profiles demonstrating epitope-specific α-Syn immunolabelling in neuronal and oligodendroglial inclusions in MSA at different Z-stack levels. (a) Epitope-specific immunolabelling of discrete interlacing α-Syn filaments in a neuron. (b-d) Epitope-specific immunolabelling of discrete interlacing α-Syn filaments in representative oligodendroglia. A distinct C-terminus immunopositive periphery encapsulating inclusions is evident in (b) and (c). The accompanying fluorescent profiles in each image validate the epitope-specific α-Syn immunolabelling of discrete α-Syn filaments. Accompanying Supplementary Profile Videos are presented using the same colour scheme.

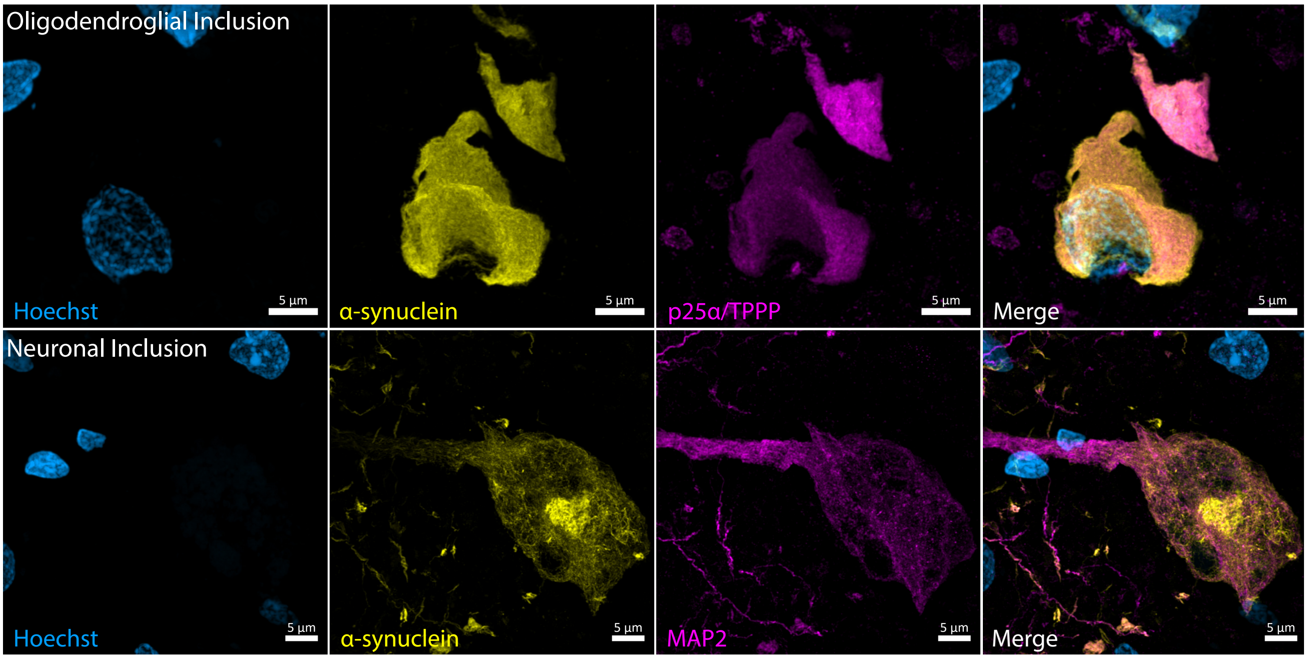

Fig. S8 Morphological demarcation of oligodendroglial and neuronal α-synuclein inclusions by the cellular periphery.

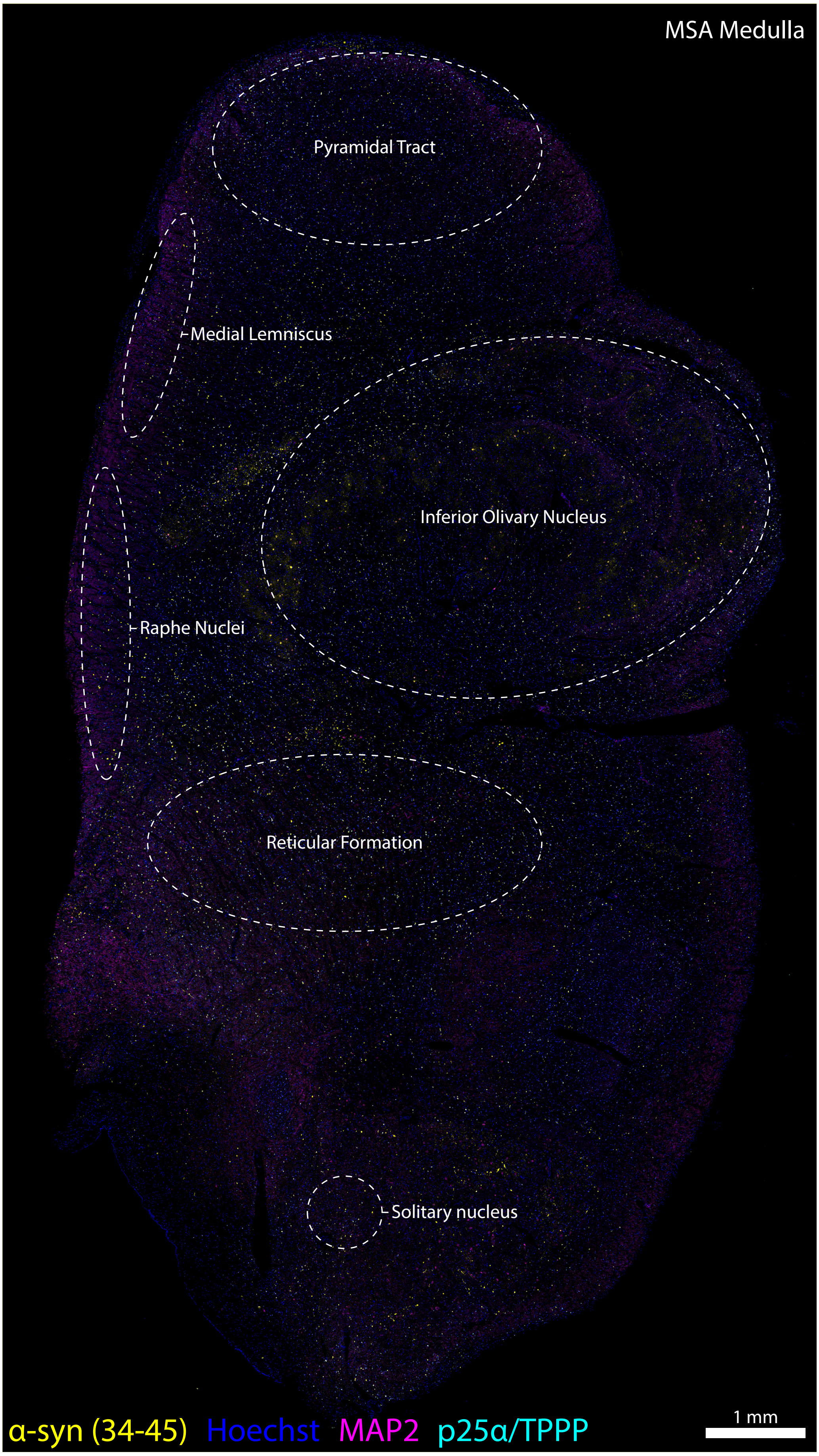

Fig. S9 Distribution of α-Syn pathology (yellow) in the MSA medulla. Dashed annotations indicate the approximate anatomical locations of the pyramidal tract, medial lemniscus, inferior olivary nucleus, raphe nuclei, reticular formation, and solitary nucleus.

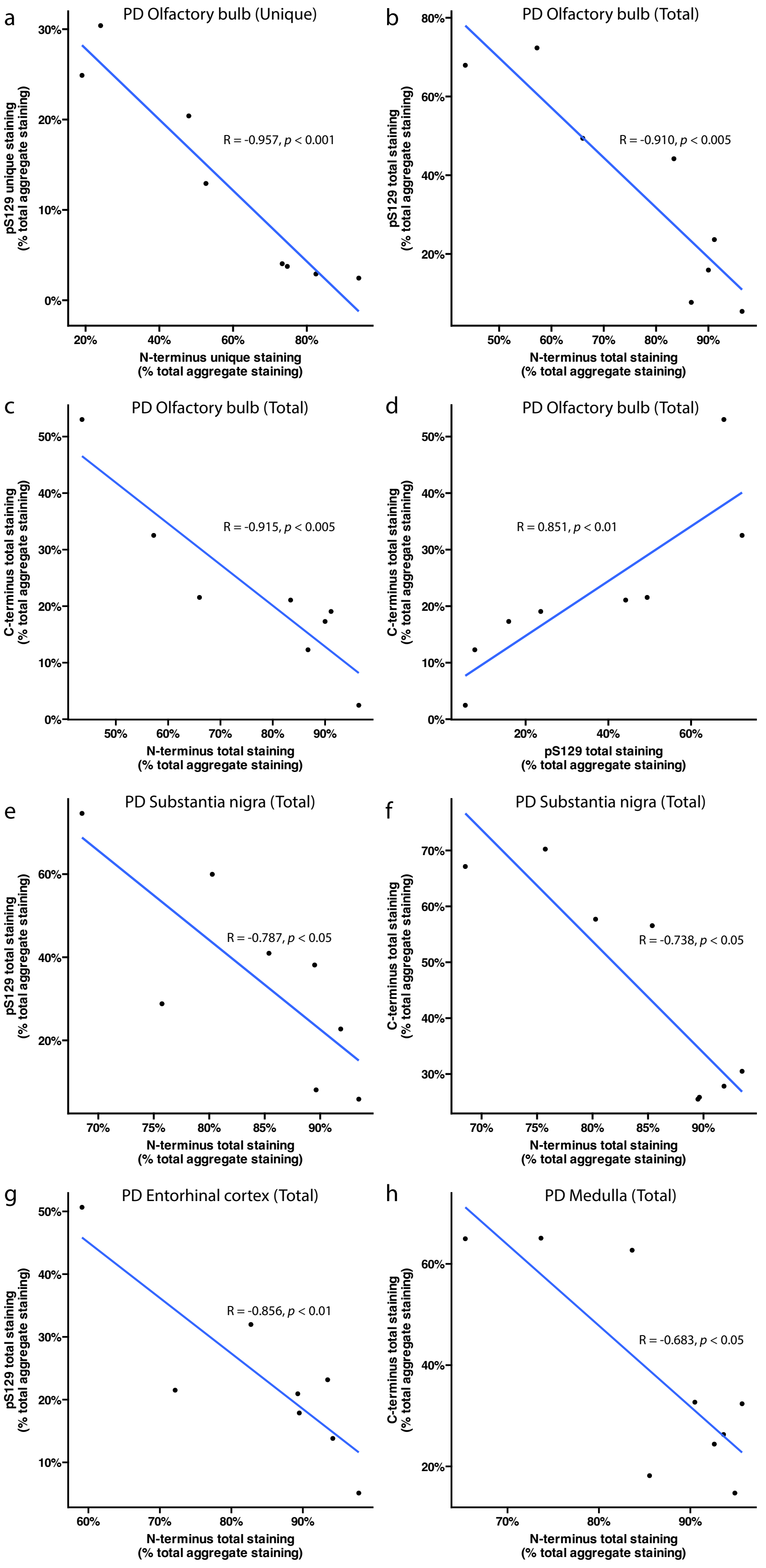

Fig. S10 Statistically significant correlations between N-terminus, pS129, and C-terminus (epitope-specific and total) immunolabelling in PD. (a) Negative correlation of *unique* N-terminus and pS129 α-Syn immunolabelling in the PD OFB. (b) Negative correlation of *total* N-terminus and pS129 immunolabelling, and (c) total N-terminus and C-terminus α-Syn immunolabelling in the PD OFB. (d) Positive correlation of total C-terminus and pS129 α-Syn immunolabelling in the PD OFB. Negative correlation of (e) total N-terminus and pS129 α-Syn immunolabelling, and (f) total N-terminus and C-terminus α-Syn immunolabelling in the PD substantia nigra. (g) Negative correlation of total N-terminus and pS129 α-Syn immunolabelling in the PD entorhinal cortex. (h) Negative correlation of total N-terminus and C-terminus α-Syn immunolabelling in the PD medulla.

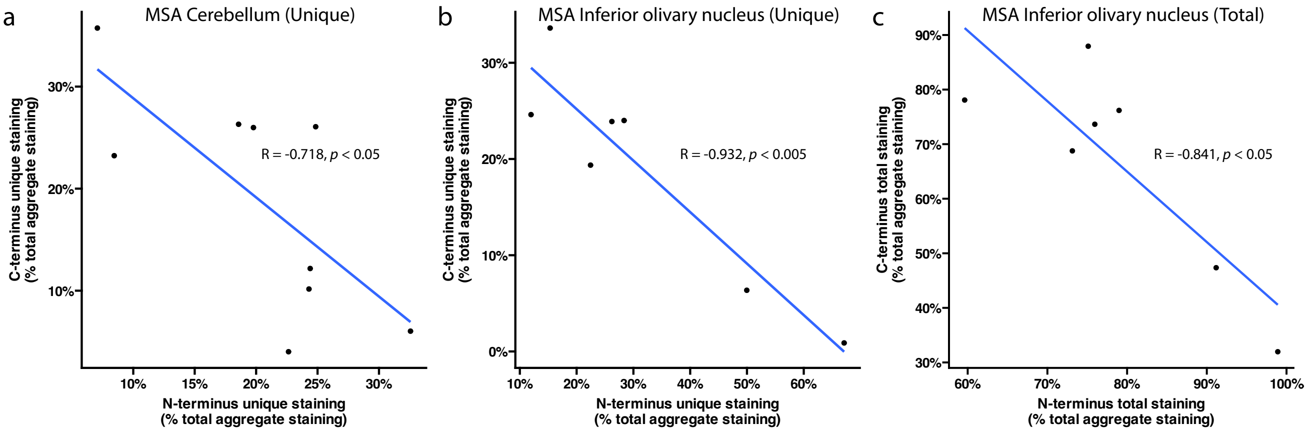

Fig. S11 Statistically significant correlations between N-terminus, pS129, and C-terminus (epitope-specific and total) immunolabelling in MSA. (a) Negative correlation of *unique* N-terminus and pS129 α-Syn immunolabelling in the MSA cerebellum. (b) Negative correlation of *unique* N-terminus and C-terminus immunolabelling, and (c) *total* N-terminus and C-terminus α-Syn immunolabelling in the MSA inferior olivary nucleus.

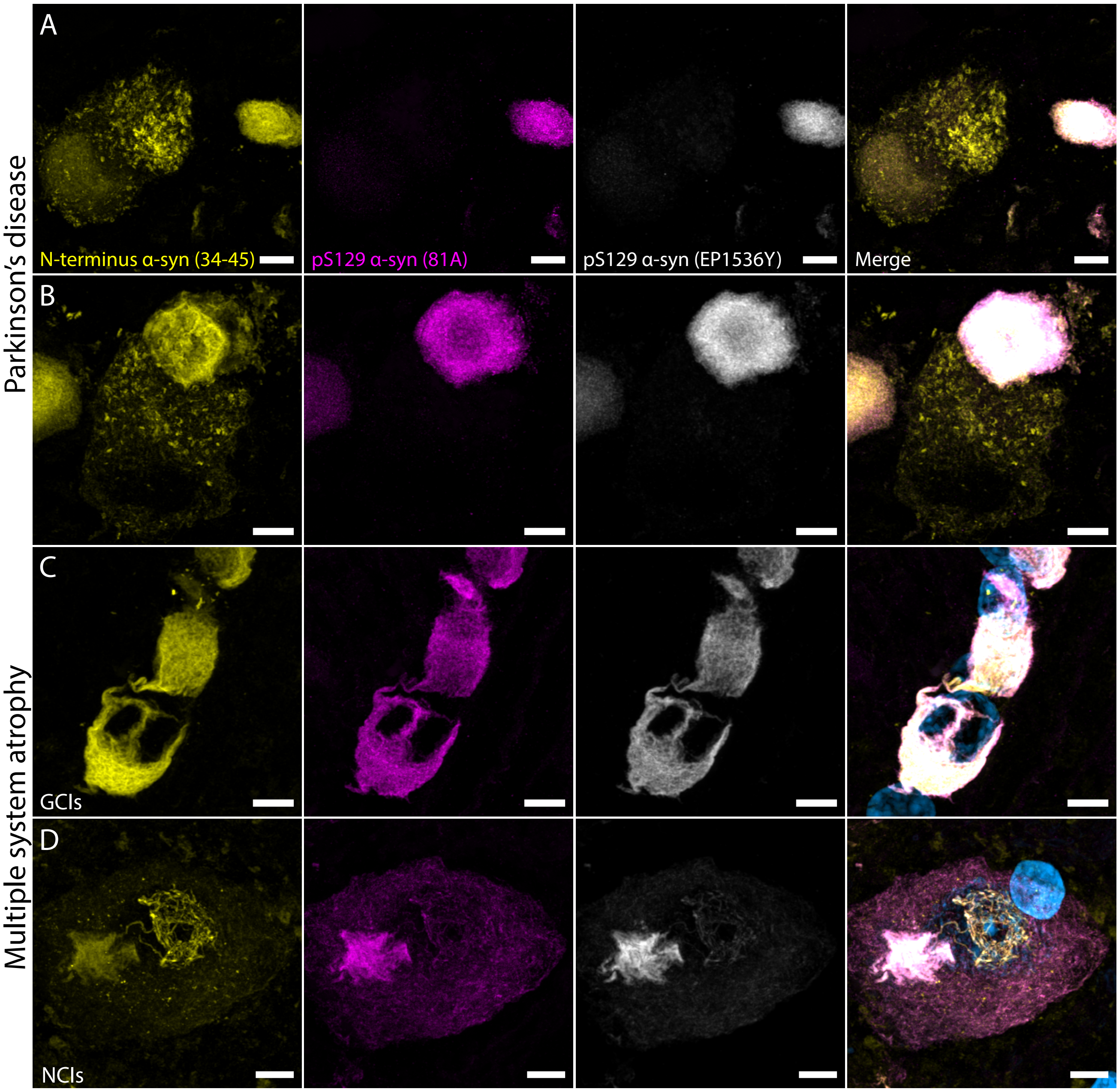

Fig. S12 Representative single channel and merged confocal images depicting the comparable immunolabelling profile of the ab184674 (81A) and ab51253 (EP1536Y) pS129 α-Syn antibodies in both PD and MSA. Scale bar, 5 µm.

References

1. Waldvogel, H. J., Curtis, M. A., Baer, K., Rees, M. I. & Faull, R. L. M. Immunohistochemical staining of post-mortem adult human brain sections. *Nature Protocols* **1**, 2719–2732 (2007).

2. Murray, H. C. *et al.* Lamina-specific immunohistochemical signatures in the olfactory bulb of healthy, Alzheimer's and Parkinson's disease patients. *Communications Biology* **5**, (2022).

3. Zapiec, B. *et al.* A ventral glomerular deficit in Parkinson's disease revealed by whole olfactory bulb reconstruction. *Brain* **140**, 2722–2736 (2017).
4. Wiseman, J. A. *et al.* Aggregate-prone brain regions in Parkinson's disease are rich in unique N-terminus α-synuclein conformers with high proteolysis susceptibility. *npj Parkinsons Dis.* **10**, 1–18 (2024).

4 Supplementary Videos

Video S1, S2, S5, S6, and S7: N-terminus α-Syn (yellow), C-terminus α-Syn (blue/navy), Hoechst (cyan).

Video S3: N-terminus α-Syn (yellow), MAP2 (blue/navy), Hoechst (cyan).

Video S4: N-terminus α-Syn (blue/navy), C-terminus α-Syn (yellow), Hoechst (cyan).

Profile Videos S1-S5: N-terminus α-Syn (yellow), C-terminus α-Syn (magenta), pS129 α-Syn (white), Hoechst (blue/cyan); where the marker is present.
